# Nucleotides as an Anti‐Aging Supplementation in Older Adults: A Randomized Controlled Trial (TALENTs study)

**DOI:** 10.1002/advs.202417728

**Published:** 2025-05-28

**Authors:** Shuyue Wang, Lixia Song, Rui Fan, Qianqian Chen, Ruisheng Fu, Mei You, Yuxiao Wu, Meng Cai, Yong Li, Meihong Xu

**Affiliations:** ^1^ Department of Nutrition and Food Hygiene School of Public Health Peking University Beijing 100191 China; ^2^ Beijing Key Laboratory of Toxicological Research and Risk Assessment for Food Safety Peking University Beijing 100191 China

**Keywords:** anti‐aging intervention, body composition, DNA methylation age, insulin sensitivity, nucleotides supplementation

## Abstract

Aging impairs nutrient metabolism and accelerates biological aging, negatively affecting health and longevity. The Targeting Aging and Longevity with Exogenous Nucleotides (TALENTs) trial (ClinicalTrials.gov: NCT05243108) aimed to explore whether nucleotides (NTs) supplementation can delay biological aging and improve health outcomes in the elderly. The trial is a 19‐week, double‐blind, randomized, placebo‐controlled study in Chengdu, China, with 121 participants (60–70 years). Participants are randomly assigned to either NTs (1.2 g day^−1^) or placebo group (1:1). The results of primary outcomes showed that NTs had significantly greater reduction in median DNA methylation age compared to placebo over 19 weeks (β = −3.08 years, 95% CI: −5.07 to −1.10, *P* = 0.0023), with a trend toward reduction observed over 11 weeks (β = −1.94 years, 95% CI: −4.32 to 0.45, *P *= 0.11); whereas no significant difference changes of leukocyte telomere length are showed between groups (week 11: β = 0.09, 95% CI: −0.10 to 0.29, *P* = 0.36; week 19: β = 0.12, 95% CI: ‐0.05 to 0.28, *P* = 0.18). Insulin sensitivity improved in the NTs group, with a significant reduction in HOMA‐IR over 19 weeks (β = −0.45, 95% CI: −0.86 to −0.04, *P* = 0.033). No severe adverse events or significant changes in safety indicators are reported. Together, our findings establish that NTs may delay biological aging and improve insulin sensitivity with a well‐tolerated safety profile.

## Graphical abstract

The TALENTs randomized controlled trial evaluates the potential of nucleotides supplementation as an anti‐aging intervention in older adults. After 19 weeks, nucleotides supplementation significantly reduces DNA methylation age, suggesting a delay in epigenetic aging, and improves insulin sensitivity without severe adverse events. These findings provide preliminary evidence for the role of nucleotides in modulating biological aging processes.

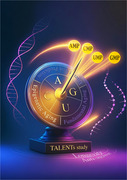

## 1 Introduction

With the global trend of population aging accelerating, extending a healthy lifespan has become a key priority in public health and clinical care. Aging is a major risk factor for many chronic diseases and functional decline, leading to a cycle of inadequate nutrient intake,^[^ ^1^ ^]^ and accelerated biological aging and raises the risk of age‐related diseases.^[^ ^2^, ^3^ ^]^ As proposed by the geroscience theory, interventions that target the underlying biological mechanisms of aging, rather than individual diseases, have emerged as promising strategies to improve long‐term health outcomes and quality of life in older adults.^[^ ^4^, ^5^ ^]^

Food‐derived nutritional interventions are safe, multifunctional, and cost‐effective, making them attractive options for promoting health and longevity. Nucleotides (NTs), as essential components of DNA and RNA, play a critical role in cellular repair, energy metabolism, and genomic stability.^[^ ^6^ ^]^ However, as the body ages, its ability to synthesize and absorb exogenous nucleotides declines, leading to a relative insufficiency that can impair these vital processes.^[^ ^7^, ^8^ ^]^ Nucleotides supplementation is therefore considered a promising strategy to address this deficiency and support healthy aging. Previous animal and cellular studies have shown NTs to be safe,^[^ ^9^, ^10^, ^11^, ^12^ ^]^ with demonstrated benefits such as anti‐aging effects,^[^ ^13^, ^14^, ^15^, ^16^ ^]^ as well as improvements in metabolism^[^ ^17^, ^18^, ^19^ ^]^ and immunoregulation.^[^ ^21^, ^22^ ^]^ These findings suggest that NTs have the potential to delay aging and improve age‐related health outcomes. However, clinical trials are needed to confirm these effects in human populations and establish their efficacy.

Although animal and in vitro studies have reported beneficial effects of NTs on aging‐related pathways, evidence from well‐controlled human studies remains limited. To address this gap, the Targeting Aging and Longevity with Exogenous Nucleotides (TALENTs) trial was designed as a randomized controlled study to evaluate the effects of NTs supplementation in older adults. To assess the biological impact of NTs, the study employed two widely recognized aging biomarkers:^[^ ^23^ ^]^ DNA methylation age (DNAmAge) and leukocyte telomere length (LTL). DNAmAge is a molecular indicator derived from age‐associated DNA methylation patterns, shown to predict multiple clinical outcomes including frailty, cardiovascular disease, diabetes, cognitive decline, and mortality.^[^ ^24^, ^25^ ^]^ LTL, a measure of telomere erosion with age and stress, reflects cellular senescence and has also been linked to age‐related disease risk and longevity.^[^ ^26^, ^27^ ^]^ Both biomarkers are increasingly used in translational aging research, and their potential for monitoring intervention effects in clinical and community settings has been demonstrated in large epidemiological cohorts and intervention trials.

In this trial, DNAmAge and LTL were pre‐specified as core endpoints to quantify biological aging and evaluate the potential of NTs to modulate age‐related molecular trajectories. Additional outcomes, including metabolic, immunological, and body composition parameters, were included to explore broader health effects and mechanisms. The results aim to contribute to the development of evidence‐based, nutrition‐oriented strategies for promoting healthy aging in the older population.

## 2 Result

### 2.1 Participants

**Figure 1:**
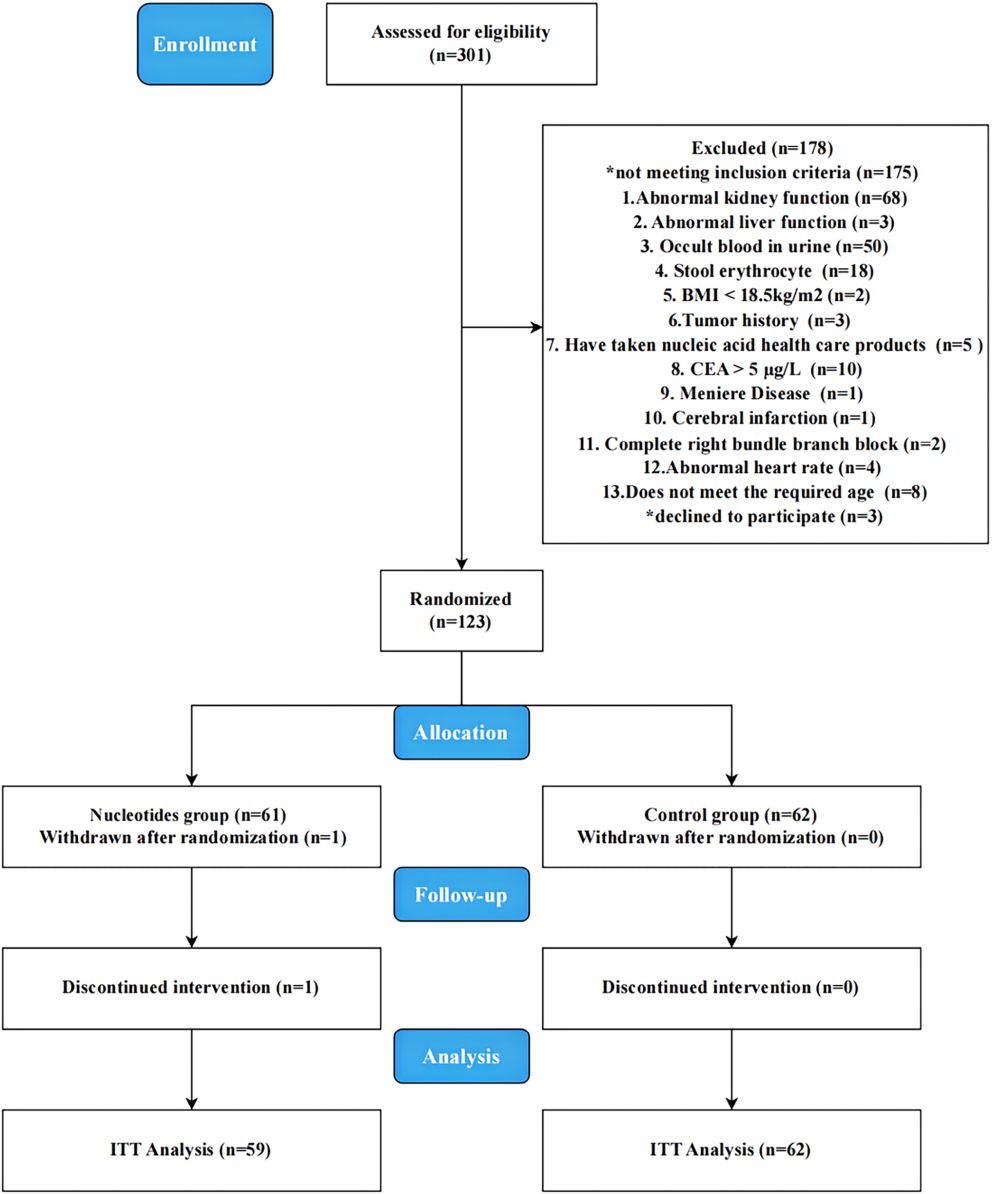
Consort diagram for the TALENTs Trial.

During the period from August 23, 2022 to October 15, 2022, a total of 301 elderly people in the Chengdu community who were willing to participate and signed informed consent were recruited, and 123 subjects were finally determined to be eligible for participation after the screening. The most common reasons for exclusion were noncompliance with inclusion criteria (*n* = 175). Prior to the intervention, one participant in the nucleotides group withdrew. The intervention commenced on October 23, 2022, and concluded on March 8, 2023, spanning 19 weeks. During the follow‐up period, aside from one participant in the NTs group who withdrew after a traffic accident one week into the intervention, the remaining 121 participants completed the trial. **Figure** **1** shows the flow chart of the study samples. Consequently, a total of 121 participants who completed the intervention were included in the final statistical analysis. A summary of 121 participant demographics at baseline is presented in **Table** **1**. There were no significant differences between groups for any baseline characteristics. The mean age was 65.65 ([SD] 2.59) years, and 82 (67.21%) were women.

**Table 1:** Characteristics of TALENTs Trial participants at baseline.

	Overall [n = 121]	Nucleotides Group [n = 59]	Control Group [*n* = 62]	*p*
**Age, years; mean±SD**	65.65 ± 2.59	65.55 ± 2.63	65.74 ± 2.58	0.685
**Female, n (%)**	80(66.12)	39(66.10)	41(66.13)	0.997
**Nationality, n (%)**				
Han nationality	120(99.17)	58(98.30)	62(100)	0.488
**Marital status, n (%)**				
single	2(1.65)	2(3.39)	0(0)	0.452
married	102(84.30)	49(83.05)	53(85.48)	
divorced/widowed	17(14.05)	8(13.56)	9(14.52)	
**Education level, n (%)**				
primary school and below	5(4.13)	3(5.08)	2(3.23)	0.116
Junior high school	45(37.19)	25(42.37)	20(32.26)	
High school/technical secondary school	41(33.88)	22(37.29)	19(30.65)	
University/college or above	30(24.79)	9(15.25)	21(33.87)	
**Smoking status, n (%)**				
Never smoked	106(87.60)	48(81.36)	58(93.55)	0.135
Have quit smoking	7(5.79)	5(8.47)	2(3.23)	
smoking	8(6.61)	6(10.17)	2(3.23)	
**Alcohol consumption, n (%)**				
Never drank alcohol	98(80.99)	47(79.66)	51(82.26)	0.676
Have stopped drinking	6(4.96)	4(6.78)	2(3.23)	
drinking	17(14.05)	8(13.56)	9(14.52)	
**Comorbidities, n (%)**				
Hypertension	36(29.51)	20(33.33)	16(25.81)	0.362
Dyslipidemia	10(8.20)	2(3.33)	8(12.90)	0.095
Diabetes	17(13.93)	9(15.00)	8(12.90)	0.738
Cardiovascular disease	66(54.10)	37(61.67)	29(46.77)	0.099
Chronic respiratory diseases	40(32.79)	22(36.67)	18(29.03)	0.369
Fatty liver	45(36.89)	22(36.67)	23(37.10)	0.961
Renal disease	49(40.16)	20(33.33)	29(46.77)	0.130
**Number of drug types (types)**				
0	95(78.51)	45(76.27)	50(80.65)	0.967
1	13(38.52)	7(11.86)	6(9.68)	
2	4(10.74)	2(3.39)	2(3.23)	
3	4(10.74)	2(3.39)	2(3.23)	
≥4	5(4.13)	3(5.08)	2(3.23)	

Data are mean±SD or n (%). The *p*‐value is calculated through a t‐test or chi‐square test.

During the study, a COVID‐19 outbreak occurred, coinciding with the midpoint of the intervention. By the 11‐week assessment, 89 participants had contracted COVID‐19, with 42 cases in the intervention group and 47 in the control group. By the end of the intervention, a total of 93 participants reported COVID‐19 infection—44 in the intervention group and 49 in the control group—indicating no significant difference in infection rates between the groups (Table S1, Supporting Information). Regarding adherence, two participants briefly discontinued the intervention due to viral infections, with interruptions of 1 and 4 days, respectively, both accounting for less than 5% of the total trial duration. Overall, the remaining participants demonstrated high adherence to the intervention protocol. Additionally, the intake of the three major macronutrients, dietary nucleotides, and purines remained balanced between the two groups before and after the intervention (Table S2, Supporting Information).

### 2.2 Primary Outcome

**Figure 2:**
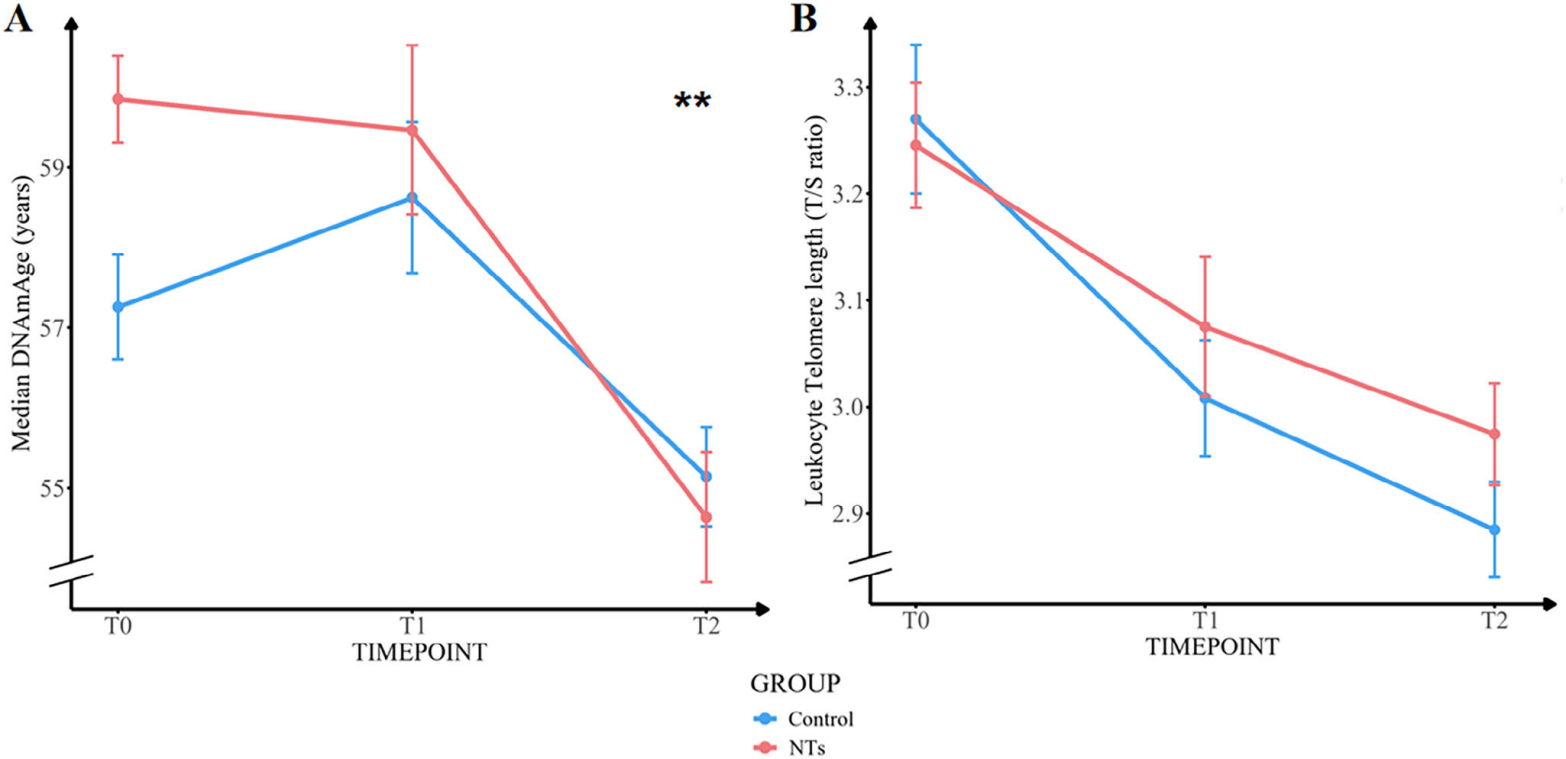
DNA methylation age and leukocyte telomere length from baseline to 11‐ and 19‐week follow‐up in the NTs and Control groups.

The NTs intervention led to a significant reduction in Median DNAmAge over the intervention period (19 weeks). The NTs group exhibited a differential change of β = −3.08 years versus control over 19 weeks (95% CI: −5.07 to −1.10, *p* = 0.0023 for Model 1), indicating a substantial decrease in biological aging markers. This significant reduction was consistent across all models (*p *< 0.01 for Model 2 and Model 3) (**Figure** **2**A, Tables S3–S4, Supporting Information). In contrast, no significant between‐group difference was observed in LTL (T/S ratio) change over time, with a mean difference of β = 0.11 (95% CI: −0.06 to 0.28, *p* = 0.19 for Model 1, *p* = 0.20 for Model 2, and *p* = 0.19 for Model 3), indicating that the NTs did not have a noticeable effect on telomere length across all models (Figure 2B, Tables S3–S4, Supporting Information).

NTs refer to nucleotides supplementation. Panel A illustrates the impact of NTs on median DNAmAge (years) and Panel B illustrates the impact of NTs on leukocyte telomere length (T/S ratio) over time in the Control group (blue) and NTs group (red), with data shown as Mean±SD. The *p*‐value signifies the statistical significance of the group‐time interaction term in generalized estimating equations (GEE) model 1. **p  <  0.01. Panel A demonstrates a significant reduction in DNA methylation age in the NTs group compared to the Control group (*p* = 0.0023). Panel B shows no significant difference in telomere length between the two groups (*p* = 0.19). T0 refers to the baseline measurement, T1 represents the midpoint (week 11), and T2 indicates the endpoint (week 19). Sample sizes (T0/T1/T2): Leukocyte Telomere length ‐59/57/57 (NTs), 62/61/61 (Control); DNA methylation age ‐59/41/57 (NTs), 62/42/61 (Control).

The sensitivity analysis, incorporating midpoint measurement data into the generalized estimating equations (GEE) model with the same covariates, confirmed that the primary results on Median DNAmAge and LTL change over time remained consistent (Table S5, Supporting Information). At the mid‐term intervention point (11 weeks), the results for Median DNAmAge showed a similar trend. The NTs group exhibited a differential change of β = −1.94 years versus control (95% CI: −4.32 to 0.45, *p* = 0.11 for all models). Although this reduction did not reach statistical significance, it indicated a potential trend toward a decrease in biological aging markers, which was consistent with the observed effect at the 19‐week endpoint. No significant changes were observed in LTL (T/S ratio) at 11 weeks either, with β = 0.09 (95% CI: −0.10 to 0.29, *p* = 0.36 for all models) (Table S5, Supporting Information).

Notably, COVID‐19 infections during the trial period may have transiently accelerated aging‐related processes around the midpoint (T1). This was reflected in a general reduction in LTL observed in both groups (NTs: −0.18 ± 0.54; Control: ‐0.27 ± 0.57), although the between‐group difference was not statistically significant (*p* = 0.36, *Cohen's d* = 0.17). In contrast, changes in median DNAmAge at T1 exhibited opposite trends between groups. While the control group showed an increase in DNAmAge, the NTs group demonstrated a reduction (NTs: −1.03 ± 5.24; Control: 1.30 ± 6.48). Although this difference did not reach statistical significance (*p* = 0.07, *Cohen's d* = −0.40), the divergence in direction is noteworthy and suggests a potential intervention effect rather than a regression to the mean (Figure 2; Table S3, Supporting Information).

### 2.3 Secondary Outcome

**Figure 3:**
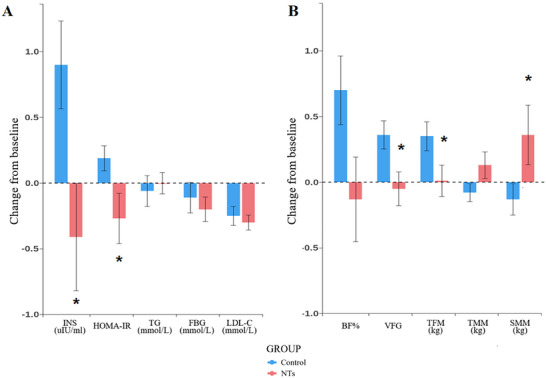
Change of Glycolipid metabolic profile and Body composition from baseline to 19‐week follow‐up in the NTs and Control groups.

For the glycolipid metabolic profile, compared with the control group, Homeostatic Model Assessment (HOMA‐IR) demonstrated a significantly greater decrease in NTs group than control over 19 weeks, indicating improved insulin sensitivity, with β = −0.45 (95% CI: −0.86 to −0.04, *p* = 0.033 for Model 1 and Model 3; *p* = 0.034 for Model 2). Similarly, insulin (INS) levels showed a significantly greater reduction, with β = −1.28 (95% CI: −2.30 to −0.26, *p* = 0.014 for all models). However, the following variables showed no significant changes: fasting blood glucose (FBG), glycated hemoglobin (HbA1c), total cholesterol (TC), triglycerides (TG) and Low‐Density Lipoprotein Cholesterol (LDL‐C) (*p *> 0.05 for all models) (**Figure** **3**A; Tables S3–S4, Supporting Information).

The NTs intervention did not lead to significant changes in T lymphocyte subsets. The following variables remained non‐significant across all models: CD3+ (%), CD3+CD4+ (%), CD3+CD8+ (%) (all *p* > 0.05 for Models 1, 2, and 3). The CD4+/CD8+ ratio showed an improvement trend, with β = 0.14 (95% CI: −0.01 to 0.30, *p* = 0.061 for all Models), indicating a potential effect of the intervention on immune regulation, though the result did not reach statistical significance. In the category of inflammatory cytokine, oxidative stress, and gene stability, none of the variables exhibited significant changes. Tumor Necrosis Factor‐alpha (TNF‐α), Interleukin‐6 (IL‐6) Gamma‐H2A Histone Family Member X, malondialdehyde (MDA), and γH_2_AX were all non‐significant (*p* > 0.05 for all models) (Tables S3–S4, Supporting Information). Sensitivity analysis indicated consistent results (Table S5, Supporting Information).

NTs refer to nucleotides supplementation. Panel A illustrates the impact of NTs on Glycolipid metabolic profile and Panel B illustrates the impact of NTs on Body composition in the Control group (blue) and NTs group (red), with data of change from baseline to week 19 depicted using vertical bar charts. The *P*‐value signifies the statistical significance of the group‐time interaction term in generalized estimating equations (GEE) model 1. *p  <  0.05. Panel A demonstrates a significant reduction in INS and HOMA‐IR in the NTs group compared to the Control group (*p* = 0.014, *p* = 0.033). Panel B demonstrates a significant increase in SMM and reduction in VFG and TFM in the NTs group compared to the Control group (*p* = 0.015, *p* = 0.044, *p* = 0.046). INS: Insulin, HOMA‐IR: Homeostasis Model Assessment of Insulin Resistance, TG: Triglycerides, FBG: Fasting Blood Glucose, LDL‐C: Low‐Density Lipoprotein Cholesterol, BF%: Body Fat Percentage, VFG: Visceral Fat Grade, TFM: Trunk Fat Mass, TMM: Trunk Muscle Mass, SMM: Skeletal Muscle Mass. Sample sizes (T0/T2): Glycolipid metabolic profile ‐59/57 (NTs), 62/61 (Control); Body composition – 58/57 (NTs), 62/61 (Control).

### 2.4 Other Outcome

In terms of body composition, compared with the control group, the NTs group showed an increase in Skeletal Muscle Mass (SMM), with β = 0.50 kg (95% CI: 0.01 to 1.00, *p* = 0.046 for all Models). Trunk Muscle Mass (TMM) also showed a trend toward improvement, with β = 0.22 kg (95% CI: ‐0.02 to 0.45, *p* = 0.072 for all models. Meanwhile, the NTs intervention led to significant reductions in Visceral Fat Grade (VFG), with β = −0.40 (95% CI: −0.73 to −0.08, *p* = 0.015 for all Models), and Trunk Fat Mass (TFM), which was reduced by β = −0.33 kg (95% CI: −0.64 to ‐0.01, *p* = 0.044 for Model 1 and 3; *p* = 0.046 for Models 2). Additionally, the Body Fat Rate (BFR) in the NTs group showed a trend toward a reduction, with β = −0.80% (95% CI: −1.61 to 0.00, *p* = 0.05 for Model 1 and 3; *p* = 0.052 for Models 2) (Figure 3B; Tables S6–S7, Supporting Information).

For the comprehensive geriatric questionnaire assessment (CGA), the following variables were all non‐significant (*P* > 0.05 for all models): the Frailty Index, the Short Form‐12 Health Survey (SF‐12), the Montreal Cognitive Assessment (MoCA), the Pittsburgh Sleep Quality Index (PSQI), and the Kessler Psychological Distress Scale (K10). This indicates no measurable impact of the NTs on these aspects of cognitive function, emotional well‐being, or overall quality of life. For fatigue, as measured by the Fatigue Scale‐14, significantly improved, with β = −1.41 (95% CI: −2.54 to −0.28, *p* = 0.014 for Models 1 and 3; *p* = 0.015 for Models 2). Additionally, overall physical fatigue was significantly reduced, with β = −0.93 (95% CI: −1.67 to −0.19, *p* = 0.014 for all models) (Tables S6–S7, Supporting Information).

### 2.5 Safety

The safety analysis revealed no statistically significant differences in adverse events between the NTs and control groups. The most commonly reported symptoms, including gastrointestinal discomfort and sleep disturbances, predominantly occurred in the early phase of the intervention, with a notable decrease in frequency over time. Tumor biomarkers, Carcinoembryonic Antigen (CEA), and Alpha‐Fetoprotein (AFP) demonstrated no significant differences between groups across all models. Liver and kidney function parameters, including Alanine Aminotransferase (ALT), Aspartate Aminotransferase (AST), and Alkaline Phosphatase (ALP), remained unchanged throughout the study.  The NTs group showed significantly elevated uric acid (UA) over 11 weeks (β = 31.61 µmol/L, 95%CI:16.15 to 47.07, *p* < 0.001) versus controls, with the between‐group change attenuating to non‐significance by 19 weeks (β = 14.39 µmol/L, 95%CI:‐2.79 to 31.57, *p* = 0.10). This temporal pattern was consistent across all models. No significant differences were observed in renal function markers (cystatin C, β2‐microglobulin, urinary albumin) or hematological parameters (Red Blood Cell, White Blood Cell, lymphocyte/neutrophil counts) between groups. Overall, these findings support the safety of NTs (Tables S8–S11, Supporting Information).

## 3 Discussion

This randomized controlled trial demonstrated that a 19‐week course of NTs supplementation led to statistically and clinically relevant improvements in several aging‐related parameters among healthy older adults. To our knowledge, this is the first RCT to evaluate the effects of NTs supplementation in this population, underscoring the novelty and potential translational value of the findings. Notably, the intervention was associated with a reduction in DNA methylation age, suggesting a possible rejuvenation of the epigenetic clock, and showed favorable trends in insulin sensitivity. Improvements in body composition were also observed, including increased muscle mass and reductions in trunk and visceral fat. Importantly, NTs supplementation was well‐tolerated, with no significant adverse events reported, supporting its preliminary safety profile in this context.

In this trial, we selected DNAmAge and LTL as molecular biomarkers of aging to evaluate the biological effects of NTs supplementation. As described in the Introduction, both markers have been widely applied in aging research and are associated with age‐related health outcomes. Specifically, we used a composite panel of DNA methylation clocks and selected median DNAmAge as the main molecular endpoint, based on its reported ability to detect age reversal within relatively short timeframes.^[^ ^28^, ^29^ ^]^ Our results showed a statistically significant 3.08‐year reduction in median DNAmAge in the NTs group compared to placebo after 19 weeks of supplementation, suggesting a potential slowing of epigenetic aging. These findings highlight the relevance of NTs as a promising nutritional strategy, complementing other approaches such as dietary modifications, physical activity,^[^ ^30^, ^31^ ^]^ and supplementation with bioactive compounds like α‐ketoglutarate and polyphenols.^[^ ^32^, ^33^ ^]^ This change is not only statistically significant but also biologically relevant. Prior cohort studies have shown that each one‐year increase in epigenetic age acceleration is associated with a higher risk of all‐cause mortality, as well as increased incidence of cardiovascular disease, cognitive impairment, and metabolic dysfunction.^[^ ^34^ ^]^ Specifically, a meta‐analysis involving four elderly cohorts found that individuals with a 5‐year increase in Δage (difference between DNAmAge and chronological age) exhibited a 21% greater likelihood of death, even after controlling for age and sex.^[^ ^34^, ^35^ ^]^ In parallel, findings from twin studies indicated that each additional 5 years of epigenetic aging corresponds to a 35% increase in mortality risk, reinforcing the prognostic value of DNAm age independent of genetic background.^[^ ^36^ ^]^ Therefore, this degree of age reversal may reflect a meaningful biological benefit. Although DNAmAge is not yet widely used in clinical settings, it has emerged as a sensitive and dynamic biomarker for tracking biological aging in intervention studies. Our findings suggest that NTs supplementation may offer a practical strategy to delay biological aging and support healthy aging in older adults. In contrast, no significant group differences were detected in LTL. This may be due to the relatively short duration of the study, as telomere attrition is generally a slower process. Prior studies reporting LTL improvement often involved longer interventions (≥24 months) and broader lifestyle modifications.^[^ ^37^, ^38^ ^]^ Moreover, short‐term changes in LTL remain controversial in the field.^[^ ^39^ ^]^ Thus, large‐scale, long‐term studies are needed to confirm the effect of NTs on LTL. Several contextual factors may have influenced the outcomes. First, the study population consisted of generally healthy older adults, potentially underestimating the observable benefits of NTs. Second, the trial was conducted during the COVID‐19 pandemic. At the midpoint (T1), both groups exhibited reductions in LTL, suggesting accelerated cellular aging under pandemic‐related stress.^[^ ^40^, ^41^ ^]^ Notably, median DNAmAge increased in the control group but decreased in the NTs group at the same time point. Although this difference did not reach statistical significance (*p* = 0.07), the contrasting trends suggest a possible protective effect of NTs against infection‐related biological aging, rather than a simple regression to the mean.

In addition to slowing biological aging, NTs supplementation was associated with improvements in metabolic health. Significant reductions in INS and HOMA‐IR were observed in the NTs group, aligning with prior cellular and animal studies reporting the beneficial effects of NTs on insulin sensitivity.^[^ ^42^, ^43^ ^]^ The intervention also improved body composition, evidenced by increased skeletal muscle mass (0.5 kg) and reductions in trunk fat mass (0.33 kg) and visceral fat grade (0.4), which may have contributed to enhanced insulin sensitivity. These findings suggest that NTs may support metabolic homeostasis by enhancing cellular energy metabolism and optimizing glucose utilization in liver and muscle tissues, thereby reducing insulin demand. Notably, insulin resistance has been linked to accelerated biological aging through its impact on DNA methylation patterns and epigenetic clocks.^[^ ^44^, ^45^ ^]^ Individuals with higher insulin resistance often show faster epigenetic aging, likely due to metabolic stress and chronic low‐grade inflammation. Therefore, the observed improvement in insulin sensitivity may partly explain the reduction in DNAmAge. While no significant changes were found in TNF‐α or MDA levels, the metabolic benefits of NTs may involve alternative pathways beyond conventional inflammatory or oxidative stress mechanisms. This potential mechanistic link between insulin regulation and epigenetic aging adds to the growing body of evidence supporting NTs as a candidate for aging‐related interventions. Nonetheless, given the exploratory outcomes and relatively small sample size, these findings should be interpreted with caution. Further studies are needed to confirm the effects of NTs on metabolic health and epigenetic aging in broader populations and over longer intervention periods.

For the safety assessment, no adverse events were observed throughout the trial. Additionally, our trial confirmed the safety of NTs across key physiological parameters, including liver and kidney function, tumor biomarkers, and routine blood parameters, with the exception of a transient increase in uric acid. This increase was likely due to accelerated purine metabolism observed at 11‐week, possibly exacerbated by COVID‐19 infections. Uric acid levels returned to baseline by 19‐week, indicating restored metabolic balance. Overall, these findings strongly support the safety of NTs.

This RCT possesses several methodological strengths that enhance the credibility of its findings. First, the randomized allocation of participants minimized selection bias and allowed for a controlled evaluation of NTs supplementation in healthy older adults. Robust statistical analyses, including sensitivity tests and adjustments for baseline differences and potential confounders, further reinforced the reliability of the results. The safety profile of NTs was also carefully assessed, with no significant adverse events or abnormal tumor biomarker changes observed during the intervention period. In addition, batch effects in biomarker measurements were well controlled, with unified sample processing protocols and centralized laboratory analysis, reducing potential variability. The study also benefited from high participant adherence, a low dropout rate, and effective blinding procedures, all of which contribute to its internal validity. Collectively, these design and implementation features provide a strong foundation for future larger‐scale trials aimed at confirming the efficacy of NTs in aging‐related health outcomes.

This study provides preliminary evidence for the potential anti‐aging effects of NTs; however, several limitations should be considered. First, the 19‐week intervention period was relatively short and may not fully capture the long‐term impact of NTs on aging‐related outcomes. Second, the sample size was determined based on the Technical Specifications for Testing and Evaluation of Health Food issued by the China National Health Commission, a widely accepted standard in functional food research. While the sample size was nearly sufficient power to detect significant changes in DNAmAge (α = 0.025, post hoc power = 78.96%), limited power for LTL (α = 0.025, post hoc power = 16.13%), suggesting that larger sample sizes may be needed in future studies to robustly evaluate effects on LTL and other outcomes with smaller expected effect sizes. These findings provide a useful reference for future trial design and power estimation. Third, baseline DNAmAge was not available at the time of randomization due to centralized batch analysis, preventing direct assessment of baseline comparability. Although statistical models adjusted for baseline DNAmAge, this limitation may influence interpretation. Additionally, participants were relatively healthy, as reflected by their lower biological age compared to chronological age, which may have reduced the observable effects of the intervention. Fourth, 76.86% (93/121) of participants were infected with COVID‐19 during the trial. While infection rates were balanced between groups, the pandemic may have acted as a biological stressor, temporarily accelerating aging. At the midpoint, opposite trends in DNAmAge between groups reduce the likelihood of regression to the mean and suggest a possible mitigating effect of NTs. Lastly, body composition was assessed using bioelectrical impedance analysis (BIA), which, while practical and widely used, is less precise than dual‐energy X‐ray absorptiometry (DEXA). Future studies could consider using DEXA to improve measurement accuracy.

NTs supplementation demonstrates promising potential in modulating biological aging, as evidenced by its effects on median DNAmAge, metabolic health, muscle mass, and fat distribution. These initial findings, together with their affordability and ease of use, indicate that NTs may offer a practical nutritional strategy for promoting healthy aging and improving the quality of life in older adults. However, the current evidence remains preliminary. Larger and longer‐term trials are needed to confirm the sustained efficacy and safety of NTs supplementation. In addition, mechanistic studies, particularly those employing multi‐omics approaches, are warranted to further elucidate whether improvements in insulin resistance mediate the observed delay in biological aging.

## 4 Conclusion

Taken together, the findings of this study suggest that NTs supplementation may represent a safe and potentially beneficial strategy for promoting healthy aging. The intervention was associated with a reduction in DNAmAge and improvements in insulin sensitivity, along with favorable changes in body composition, including increased muscle mass and decreased trunk and visceral fat. No serious adverse events were observed, and safety indicators remained within normal ranges, supporting the short‐term safety of NTs. While these results are encouraging, larger and longer‐term studies are needed to confirm the observed effects and to further evaluate the potential of NTs in delaying biological aging and mitigating age‐related functional decline.

## 5 Experimental Section

### Trial Design and Participants

The TALENTs trial was an exploratory, 19‐week, single‐center, double‐blind, randomized placebo‐controlled study conducted in Chengdu, Sichuan Province, China. The trial was registered at ClinicalTrials.gov (NCT05243108). Ethical approval was obtained from the Biomedical Ethics Committee of Peking University (ethics review approval number: IRB00001052‐21114, date of approval: November 17, 2021). Details of the trial design were reported previously.^[^ ^46^ ^]^

Community‐living individuals were recruited through advertisements, and a comprehensive health assessment was conducted, including clinical health physical examination, questionnaire survey, physical function assessment, and anthropometrics. Eligibility was determined based on the following criteria. Inclusion Criteria: 1) Age between 60 and 70 years; 2) No serious physical or mental illness; 3) No previous use of nucleotide‐related supplements or health foods; 4) Ability to follow the study protocol and provide informed consent. Exclusion Criteria: 1) Confirmed diseases such as autoimmune system diseases, serious cardiovascular and cerebrovascular diseases, major organ complications such as in the liver and kidney, or complicated with other serious diseases such as malignant tumors, pancreatic diseases, and mental diseases; 2) Having abnormal screening laboratory test values or other lab test results that would preclude study participation in the judgment of the investigator; 3) Severe visual or hearing impairments affecting communication; 4) Participation in other clinical trials within the last 6 months; 5) Having used food or medicine that is relevant to the function being tested. All participants provided informed written consent prior to recruitment into the study, in accordance with the Declaration of Helsinki.

### Sample Size and Randomization

The sample size for this study was determined according to the Technical Specifications for Testing and Evaluation of Health Food issued by the China National Health Commission, which recommends a minimum of 50 participants per group for human intervention trials. Participants were randomly assigned in a 1:1 ratio to the intervention or control group. To account for an estimated 20% loss to follow‐up, a total of 120 participants were enrolled.

Participants were randomly assigned to groups using computer‐generated random numbers, with the scheme known only to the study designer, who was not involved in data collection or analysis. The randomization code was sealed and would only be unblinded in cases of serious adverse events.

### Intervention and Blinding

The intervention was administered in capsule form to ensure consistency in appearance, taste, and formulation between the placebo (pure starch excipient) and the intervention (nucleotides plus starch excipient) to avoid raising any suspicion among participants. The intervention group received capsules containing exogenous nucleotides, enzymatically hydrolyzed from food yeast. Placebo capsules were designed to closely resemble the intervention capsules. Each nucleotides capsule contained 0.1 g of starch excipients and 0.3 g of nucleotides, while each placebo capsule contained 0.4 g of starch excipients. The nucleotides composition followed the ratio of 5’‐AMP:5’‐CMP:5’‐GMPNa2:5’‐UMPNa2 = 16:41:19:24, modeled after the composition in human breast milk and based on previous studies.^[^ ^47^, ^48^ ^]^ Both the nucleotides and placebo capsules were provided by Zhen‐Ao Biotechnology Co., Ltd. (Dalian, China), stored in a cool and ventilated room, with entry and exit meticulously recorded by a designated specialist. The daily dosage of 1.2 g of nucleotides fell within the range approved for commercially available health food products in China and is consistent with previous studies.^[^ ^48^, ^49^ ^]^ Participants were instructed to take four capsules daily while maintaining their regular lifestyle and dietary habits.

The double‐blind design ensured that both participants and study personnel—including investigators, clinicians, data collectors, and follow‐up coordinators—remained unaware of group assignments. Capsules were packaged in opaque bottles labeled only with participant identifiers. Blinding procedures were thoroughly explained to participants during the informed consent process.

### Outcomes

The primary outcomes of this study were DNAmAge and TLT, which serve as indicators of biological aging and cellular senescence. The median of four principal component (PC)‐corrected epigenetic ages (Horvath,^[^ ^50^ ^]^ Hannum,^[^ ^51^ ^]^ GrimAge,^[^ ^52^ ^]^ and DNAm PhenoAge^[^ ^53^ ^]^) was generated for each participant and presented as a value called “Median DNAmAge”. The Median DNAmAge mitigates specific errors of individual clocks, enhancing overall accuracy and consistency in aging assessments.^[^ ^29^ ^]^ Meanwhile, our primary analysis focused on PC‐corrected versions, which provide improved biological age estimates by controlling for confounders and reducing noise).^[^ ^54^ ^]^ All age estimates were obtained using the R package (https://github.com/yiluyucheng/dnaMethyAge) developed by Lu et al.^[^ ^55^ ^]^

Secondary outcomes included alterations in aging processes such as glycolipid metabolic profile, immune function, inflammatory cytokine levels, and oxidative and gene stability. Other outcomes comprised a CGA and body composition.

### Safety Assessments

Adverse events, such as falls, fractures, hospitalizations, and mortality, were meticulously recorded in dedicated adverse event logs, ensuring comprehensive safety and compliance evaluations throughout the follow‐up period. In addition, routine blood tests, liver and kidney function assessments, and tumor biomarker assessments were conducted to ensure safety monitoring.

### Data Collection

In the TALENTs study, data collection encompassed a comprehensive range of biological and psychological assessments, utilizing validated tools, and biochemical assays for data collection. Data and biological sample collection at baseline (T0), midpoint (T1), and endpoint (T2) were executed by well‐trained and skilled researchers. Clinical examinations and biological samples (blood, urine, and feces) were collected at Aikang Guobin Medical Examination Hospital (Chengdu, China). A full overview of outcome measures and their respective time points is detailed in Table S12 (Supporting Information). Detailed methodologies and the complete list of reagents are available in Table S13 (Supporting Information).

To minimize batch effects and measurement bias, biological samples from different time points were analyzed concurrently whenever possible. For assays requiring separate processing, normalization procedures were applied to ensure data comparability. Samples for DNA methylation, telomere length, inflammatory markers, gene stability, and oxidative stress were stored at −80 °C and analyzed simultaneously at the end of the trial. Routine clinical laboratory tests were performed immediately after collection following standardized protocols. Flow cytometry for T lymphocyte subsets was conducted on fresh samples on the day of collection. Rigorous normalization and quality control procedures were implemented during data analysis, and no significant batch effects were identified.

### Data Collection—Fasting Venous Blood Samples

Telomere length was quantified via quantitative PCR, using telomeric DNA amplification compared to a reference gene. DNA methylation data were assessed through whole‐genome bisulfite sequencing (WGBS), outsourced to BGI Genomics (Shenzhen, China) with sequencing data processed using SOAPnuke^[^ ^56^ ^]^ and Bismark^[^ ^57^ ^]^ software for filtration, alignment, and methylation mapping. The glycemic metabolic profile included FBG (measured using the glucose oxidase method), HbA1c (analyzed via high‐performance liquid chromatography), and INS (measured by chemiluminescent immunoassay). Insulin resistance was calculated using the HOMA‐IR, which is a widely used surrogate measure derived from the formula: (FBG[mmol/L]*INS [mIU/L])/22.5.^[^ ^58^ ^]^ Lipid profiles, including TC, HDL‐C, LDL‐C, and TG, were analyzed using enzymatic methods. T lymphocyte subsets (CD3+, CD4+, CD8+) were analyzed using flow cytometry, employing BD Pharmingen fluorescence‐labeled antibodies. Markers of inflammatory indicators were measured using a custom Quantibody Array (RayBiotech). Oxidative stress marker, MDA, assessed via the thiobarbituric acid method. Liver and kidney function markers, such as ALT, AST, creatinine, and cystatin C, were measured using enzymatic assays. Tumor biomarkers (CEA, AFP) were quantified via chemiluminescent immunoassay.

### Data Collection—Questionnaire‐Based Assessments

Several validated questionnaire‐based assessments were utilized, including the Frailty Index^[^ ^59^ ^]^ (assessing 28 health variables), SF‐12^[^ ^60^ ^]^ (evaluating physical and mental health), MoCA^[^ ^61^ ^]^ (screening for mild cognitive impairment), PSQI^[^ ^62^ ^]^ (measuring sleep quality), the Fatigue Scale‐14^[^ ^63^ ^]^ (assessing physical and mental fatigue), and K10^[^ ^64^ ^]^ (evaluating psychological distress). Dietary intake was captured through both 24‐hour dietary recall^[^ ^65^ ^]^ (24HR) interviews and a Food Frequency Questionnaire (FFQ), focusing on nucleotide‐rich foods. There is no unified method for assessing dietary nucleotide intake internationally, so this study calculates daily dietary NTs intake by subtracting other nitrogen‐containing substances (amino acids and B vitamins) from the total protein in food.^[^ ^6^ ^]^

### Data Collection—Body Composition Measurements

Bioelectrical Impedance Analysis(BIA) was used to measure the SMM, TMM, BF%, TFM, VFG, and other components in the human body.

### Data Collection—Follow‐up

The follow‐up was conducted by trained personnel, involving daily monitoring of capsule intake via WeChat groups and weekly calls to confirm adherence and assess health status. Additionally, bi‐weekly home visits were made to distribute capsules, record unused doses, and document adverse events. Due to the COVID‐19 pandemic, a number of participants contracted the virus around the 2‐month intervention period, which impacted on‐site assessments. Therefore, the mid‐term evaluation of the intervention was delayed from the initially planned 2 months, as specified in the protocol, to week 11. Consequently, the total intervention period was extended from the originally scheduled 4 months to 19 weeks. All participants underwent clinical examinations and biospecimen collection at week 11 and week 19; however, due to pandemic‐related restrictions, questionnaires were not conducted at week 11.

### Data Collection—Statistical Analysis

The data in this study were analyzed by intention‐to‐treat (ITT) and no interpolation was performed for data missing from general data. No data transformation, normalization, or outlier adjustment was applied in this study. Shapiro‐Wilk test was used for data normality and Levene's test for variance homogeneity.

Continuous variables were presented as mean±standard deviation(SD) for normally distributed data or median (interquartile range) for non‐normally distributed data. Categorical variables were summarized as frequencies and percentages. The sample size for each statistical analysis is represented in its respective legend and results table. The n for each analysis is based on the final available sample after accounting for missing data.

Inter‐group comparisons of pre‐intervention, post‐intervention, and change scores were performed using either independent T‐tests for normally distributed data or Mann‐Whitney U tests for non‐normally distributed data, contingent upon the distributional characteristics of the variables. *Cohen's d* was calculated using the mean difference between the two groups, with *d* = 0.2 as a small effect size, *d* = 0.5 as a medium effect size, and *d* = 0.8 as a large effect size.

We analyzed all outcomes with Generalized estimating equations (GEE) and the working correlation structure specified as exchangeable. Data from all baseline and endpoints were used. The primary model was specified as: outcome ~ group + time + sex + age + group×time interaction, where group was coded as 0 (control) and 1 (intervention), and time was coded as 0 (baseline) and 1 (endpoint). Three hierarchical models were constructed. Model 1 (base model): adjusted for sex and age. Model 2: Model 1 + dietary nucleotides intake. Model 3: Model 1 + dietary purine intake. The intervention effect was determined by the coefficient of the group×time interaction term (with its 95% CI), which quantifies the differential change in outcomes between groups from baseline to endpoint.

Sensitivity analyses were conducted by including all assessment time points (baseline, midpoint, and endpoint) data in the GEE analysis, using the same covariates as in the primary analysis. This approach aimed to evaluate the robustness of the primary, secondary, and safety outcomes and ensure that the findings remained consistent across different time points.

Statistical significance for the primary outcomes was defined as *p* < 0.025 (two‐tailed) due to the Bonferroni correction for two primary outcomes. For secondary and other outcomes, *p* < 0.05 (two‐tailed) was considered statistically significant. Statistical analyses were performed using R software (version 4.4.0), and the analysis was overseen by an experienced biostatistician. Group allocation was concealed using nonidentifying terms to ensure unbiased assessment.

## Conflict of Interest

The authors declare no conflict of interest.

## Supporting information



Supporting Information

## Data Availability

Research data are not shared.
