# Supplementary material for: Nucleotides as an Anti‐Aging Supplementation in Older Adults: A Randomized Controlled Trial (TALENTs study)
Source: Adv Sci (Weinh). 2025 May 28;12(33):2417728. doi: 10.1002/advs.202417728 (PMC12412603; doi:10.1002/advs.202417728)
Supplement: Supplementary file 1 — Supporting Information [file ADVS-12-2417728-s001.pdf]

ADVANCED SCIENCE
Open Access

Supporting Information

for *Adv. Sci.*, DOI 10.1002/advs.202417728

Nucleotides as an Anti-Aging Supplementation in Older Adults: A Randomized Controlled Trial (TALENTs study)

*Shuyue Wang, Lixia Song, Rui Fan, Qianqian Chen, Ruisheng Fu, Mei You, Yuxiao Wu, Meng Cai, Yong Li and Meihong Xu**

Supplementary Contents

Table S1. Records of COVID-19 during follow-up. 2
Table S2. Dietary intake for TALENTs participants at baseline and 19-week assessments. 3
Table S3. Primary and Secondary outcomes for TALENTs participants in the Nucleotides group and Control group at baseline, 11-week, and 19-week assessments. .6
Table S4. Generalized Estimating Equations of Primary and Secondary outcomes include baseline and 19-week. 11
Table S5. Sensitivity analysis of Primary and Secondary outcomes include baseline, 11-week, and 19-week. 13
Table S6. Other outcomes for TALENTs participants in the Nucleotides group and Control group at baseline, 11-week, and 19-week assessments. 16
Table S7. Generalized Estimating Equations of Other outcomes include baseline and 19-week. 20
Table S8. Safety indicators of Tumor biomarker, Routine examination of blood, Liver and kidney function for TALENTs participants in the Nucleotides group and Control group at baseline, 11-week, and 19-week assessments. 22
Table S9. Generalized Estimating Equations of Safety indicators include baseline and 19-week 26
Table S10. Sensitivity analysis of Safety indicators include baseline, 11-week, and 19-week. 28
Table S11. Adverse events for TALENTs participants. 30
Table S12. Outcome measures assessed in the TALENTs study. 31
Table S13. List of instruments and reagents. 34

Table S1. Records of COVID-19 during follow-up.

	Overall (n=121)	Nucleotides group (n=59)	Control group (n=62)	*P*
**Total COVID-19 infections in the trial, n (%)**				
Infection	93(76.86)	44(74.58)	49(80.33)	0.71
Non-infected	28(23.14)	15(25.42)	13(19.67)	
**COVID-19 infections by midpoint of the trial, n (%)**				
Infection	89 (73.55%)	42 (71.19%)	47 (75.81%)	0.71
Non-infected	22 (18.18%)	17 (28.81%)	15 (24.19%)	
**Type of COVID-19, n (%)**				
Asymptomatic	3(76.86)	2(74.58)	1(80.33)	
Mild	80(23.14)	40(25.42)	40(19.67)	0.16
Moderate	10(76.86)	2(74.58)	8(80.33)	

* The ***P-values*** are derived from chi-square tests comparing the proportion of COVID-19 infections and the severity of COVID-19 (asymptomatic, mild, moderate) between the nucleotides group and the control group.

* The severity of your most recent COVID-19 infection (either confirmed by a positive test or exhibiting typical symptoms of COVID-19):

1. **Asymptomatic** (positive nucleic acid or antigen test, but no symptoms).
2. **Mild** (positive nucleic acid or antigen test, primarily showing upper respiratory symptoms such as dry throat, sore throat, cough, fever, etc.).
3. **Moderate** (positive nucleic acid or antigen test, with persistent high fever >3 days and/or symptoms like cough, shortness of breath, but respiratory rate (RR) < 30 breaths/min and oxygen saturation >93% at rest. Chest imaging may show characteristic signs of COVID-19 pneumonia).

Table S2. Dietary intake for TALENTs participants at baseline and 19-week assessments.

Variable	Time	Nucleotides group (n=59)		Control group (n=62)		*P*	*Cohen s'd*
		N	Mean±SD/M (P25, P75)	N	Mean±SD/M (P25, P75)		
**Nutrients** [1,2].							
Energy (Kcal/day)	T0	59	1465.96(1099.99,1928.31)	62	1362.78(1108.35,1741.82)	0.55	0.16
	T2	59	1159.47(895.64,1614.66)	62	1257.79(880.69,1521.33)	0.57	0.13
	T2-T0	59	-291.95±836.34	62	-116.56±693.81	0.21	0.23
Protein (g/day)	T0	59	74.64(50.77,92.23)	62	64.35(49.33,82.35)	0.30	0.26
	T2	59	47.58(37.08,71.04)	62	50.43(40.69,66.50)	0.54	0.02
	T2-T0	59	-19.27(-46.43,9.44)	62	-14.15(-35.71,8.69)	0.25	0.25
Fat (g/day)	T0	59	48.39(26.64,73.12)	62	48.45(24.56,68.72)	0.71	0.1
	T2	59	44.66(28.08,63.55)	62	52.67(29.23,80.77)	0.30	0.16
	T2-T0	59	-9.68±64.91	62	1.03±50.39	0.31	0.18
Carbohydrate (g/day)	T0	59	181.65(136.68,229.52)	62	169.83(125.51,206.33)	0.50	0.07
	T2	59	150.49±52.85	62	154.48±52.98	0.68	0.08
	T2-T0	59	-29.97±82.91	62	-20.18±93.15	0.54	0.11
Nucleotide [3] (mg/day)	T0	59	457.40(0.00,1830.38)	62	0.00(0.00,1686.22)	0.12	0.08
	T2	59	684.10(0.00,1931.14)	62	1213.12(0.00,2608.53)	0.31	0.1
	T2-T0	59	0.00(-1098.34,1770.75)	62	436.61(0.00,2003.40)	0.16	0.13

Purine (mg/day)	T0	59	403.43(243.67,640.12)	62	372.39(192.69,534.47)	0.13	0.37
	T2	59	243.60(156.36,373.89)	62	262.13(197.61,378.77)	0.22	0.11
	T2-T0	59	-132.67(-389.70,43.78)	62	-72.37(-265.97,116.35)	0.12	0.37
**Seasonings**							
MSG (g/day)	T0	43	1.20(0.60,2.00)	43	2.00(0.50,3.50)	0.58	0.21
	T2	46	2.00(1.00,2.00)	40	1.86(0.48,3.00)	0.48	0.06
	T2-T0	43	0.00(-0.61,1.00)	40	0.00(-1.17,0.05)	0.04	0.22
Soy sauce (ml/day)	T0	59	0.00(-0.61,1.00)	62	0.00(-1.17,0.05)	0.04	0.22
	T2	57	2.00(1.00,5.00)	57	2.05(2.00,4.00)	0.38	0.08
	T2-T0	58	2.00(1.43,5.00)	59	2.14(1.61,5.00)	0.57	0.07
Oyster sauce (ml/day)	T0	59	0.00(-1.25,2.64)	62	0.00(-2.00,2.25)	0.97	0.1
	T2	32	0.57(0.26,1.21)	26	0.57(0.29,2.11)	0.44	0.27
	T2-T0	32	0.54(0.29,2.00)	22	0.86(0.30,2.00)	0.60	0
Thick broad-bean sauce(g/day)	T0	59	0.00(-0.21,0.08)	62	0.00(-0.19,0.00)	0.61	0.19
	T2	50	0.79(0.45,3.25)	47	1.71(0.79,3.38)	0.14	0.1
	T2-T0	48	1.57(0.66,3.00)	54	2.00(0.57,3.75)	0.66	0.08

* The calculation of dietary intake is based on two referenced studies [1,2].
* The calculation method for nucleotides is as described in the literature [1].
* **T0** refers to the baseline measurement, **T2** represents the endpoint measurement (19-week), and **T2-T0** represents the change from **T0** to **T2**.
* The ***P-values*** are derived from *t*-tests that compare the changes between Nucleotides group and Control group.
* ***Cohens'd*** is reported to quantify the effect size.
* For non-normally distributed data, the 95% confidence interval is represented as the median with the 25th percentile and the 75th percentile.

* MSG, monosodium glutamate.

References

[1] Fan R, Chen Q, Song L, et al. The Validity and Feasibility of Utilizing the Photo-Assisted Dietary Intake Assessment among College Students and Elderly Individuals in China [J]. Nutrients, 2024, 16(2): 211.

[2] Yang Y, Wang G, Pan X, et al. China Food Composition[M]. 2nd ed. Beijing: Peking University Medical Press, 2009.

[3] Ding T, Song G, Liu X, et al. Nucleotides as optimal candidates for essential nutrients in living organisms: A review [J]. Journal of Functional Foods, 2021, 82: 104498.

Table S3. Primary and Secondary outcomes for TALENTs participants in the Nucleotides group and Control group at baseline, 11-week, and 19-week assessments.

Variable	Time	Nucleotides group (n=59)		Control group (n=62)		*P*	Cohen's *d*
		N	Mean±SD	N	Mean±SD		
Primary outcomes							
Leukocyte Telomere length (T/S ratio)	T0	59	3.25 ± 0.45	62	3.27 ± 0.55	0.79	-0.05
	T1	57	3.08 ± 0.49	61	3.01 ± 0.42	0.43	0.15
	T2	57	2.97 ± 0.36	61	2.88 ± 0.35	0.17	0.25
	T1-T0	57	-0.18 ± 0.54	61	-0.27 ± 0.57	0.36	0.17
	T2-T0	57	-0.27 ± 0.40	61	-0.38 ± 0.55	0.21	0.23
	T2-T1	55	-0.08 ± 0.27	60	-0.11 ± 0.26	0.54	0.11
Median DNAmAge (years)	T0	59	59.85 ± 4.18	62	57.26 ± 5.16	0.0029	0.55
	T1	41	59.47 ± 6.77	42	58.62 ± 6.10	0.55	0.13
	T2	57	54.64 ± 6.14	61	55.14 ± 4.83	0.62	-0.09
	T1-T0	41	-1.03 ± 5.24	42	1.30 ± 6.48	0.075	-0.40
	T2-T0	57	-5.33 ± 5.76	61	-2.21 ± 5.34	0.0029	-0.56
	T2-T1	41	-4.82 ± 6.49	42	-3.62 ± 5.45	0.37	-0.20
Glycolipid metabolic profile							
HOMA-IR	T0	59	2.23 ± 1.97	62	2.24 ± 1.30	0.99	-0.00
	T1	57	2.09 ± 1.22	61	2.56 ± 1.44	0.061	-0.35
	T2	57	1.98 ± 1.04	61	2.40 ± 1.29	0.051	-0.36
	T1-T0	57	-0.11 ± 1.42	61	0.30 ± 0.75	0.055	-0.37
	T2-T0	57	-0.27 ± 1.45	61	0.19 ± 0.74	0.034	-0.41
	T2-T1	55	-0.17 ± 0.73	60	-0.12 ± 0.84	0.73	-0.06
FBG (mmol/L)	T0	59	6.13 ± 1.51	62	6.24 ± 1.81	0.73	-0.06

	T1	57	6.00 ± 1.26	61	6.45 ± 1.96	0.14	-0.27
	T2	57	5.96 ± 1.11	61	6.13 ± 1.28	0.45	-0.14
	T1-T0	57	-0.13 ± 0.66	61	0.20 ± 0.84	0.019	-0.43
	T2-T0	57	-0.20 ± 0.70	61	-0.11 ± 0.91	0.56	-0.11
	T2-T1	55	-0.09 ± 0.48	60	-0.32 ± 0.88	0.074	0.33
INS (uIU/ml)	T0	59	7.73 ± 4.41	62	7.96 ± 3.67	0.76	-0.06
	T1	57	7.69 ± 3.78	61	8.90 ± 3.88	0.089	-0.32
	T2	57	7.34 ± 3.19	61	8.75 ± 4.10	0.038	-0.38
	T1-T0	57	0.06 ± 3.59	61	0.87 ± 2.66	0.17	-0.26
	T2-T0	57	-0.41 ± 3.08	61	0.90 ± 2.61	0.014	-0.46
	T2-T1	55	-0.46 ± 2.62	60	0.00 ± 2.48	0.33	-0.18
HBA1c (%)	T0	59	6.03 ± 0.80	62	6.21 ± 1.16	0.31	-0.18
	T1	57	6.32 ± 0.87	61	6.47 ± 1.24	0.44	-0.14
	T2	57	5.82 ± 0.84	61	5.94 ± 1.05	0.5	-0.12
	T1-T0	57	0.29 ± 0.23	61	0.25 ± 0.37	0.48	0.13
	T2-T0	57	-0.21 ± 0.28	61	-0.28 ± 0.32	0.24	0.22
	T2-T1	55	-0.50 ± 0.23	60	-0.53 ± 0.33	0.57	0.10
LDL-C (mmol/L)	T0	59	2.92 ± 0.69	62	2.89 ± 0.68	0.78	0.05
	T1	57	3.09 ± 0.67	61	3.08 ± 0.77	0.96	0.01
	T2	57	2.62 ± 0.59	61	2.64 ± 0.73	0.88	-0.03
	T1-T0	57	0.16 ± 0.54	61	0.19 ± 0.61	0.77	-0.05
	T2-T0	57	-0.30 ± 0.43	61	-0.25 ± 0.57	0.65	-0.08
	T2-T1	55	-0.46 ± 0.51	60	-0.45 ± 0.58	0.89	-0.03
TC (mmol/L)	T0	59	5.34 ± 0.95	62	5.28 ± 0.93	0.73	0.06
	T1	57	5.16 ± 0.84	61	5.29 ± 0.96	0.45	-0.14

	T2	57	5.08 ± 0.92	61	5.09 ± 1.02	0.95	-0.01
	T1-T0	57	-0.17 ± 0.68	61	-0.00 ± 0.75	0.21	-0.23
	T2-T0	57	-0.26 ± 0.63	61	-0.20 ± 0.87	0.64	-0.08
	T2-T1	55	-0.09 ± 0.62	60	-0.21 ± 0.78	0.37	0.16
TG (mmol/L)	T0	59	1.30 ± 0.51	62	1.44 ± 0.70	0.23	-0.22
	T1	57	1.21 ± 0.55	61	1.40 ± 0.81	0.13	-0.28
	T2	57	1.30 ± 0.87	61	1.37 ± 0.94	0.66	-0.08
	T1-T0	57	-0.08 ± 0.43	61	-0.05 ± 0.83	0.79	-0.05
	T2-T0	57	0.00 ± 0.62	61	-0.06 ± 0.91	0.66	0.08
	T2-T1	55	0.07 ± 0.64	60	-0.01 ± 0.91	0.56	0.11
T lymphocyte subsets							
CD4+/CD8+ ratio	T0	43	1.76 ± 0.70	43	1.93 ± 1.00	0.36	-0.20
	T1	57	1.90 ± 0.82	61	1.84 ± 0.84	0.69	0.07
	T2	57	1.95 ± 0.79	61	2.00 ± 0.92	0.75	-0.06
	T1-T0	41	0.14 ± 0.39	42	-0.06 ± 0.35	0.013	0.56
	T2-T0	43	0.23 ± 0.40	43	0.08 ± 0.33	0.068	0.40
	T2-T1	55	0.07 ± 0.26	60	0.15 ± 0.34	0.15	-0.27
CD3+CD4+ (%)	T0	43	55.15 ± 10.12	43	55.18 ± 12.43	0.99	-0.00
	T1	57	55.17 ± 11.19	61	54.34 ± 10.71	0.68	0.08
	T2	57	56.87 ± 10.64	61	56.20 ± 11.29	0.74	0.06
	T1-T0	41	0.80 ± 4.05	42	-0.78 ± 4.34	0.089	0.38
	T2-T0	43	2.62 ± 4.57	43	0.99 ± 4.40	0.095	0.36
	T2-T1	55	1.64 ± 3.37	60	1.63 ± 4.61	0.99	0.00
CD3+CD8+ (%)	T0	43	34.49 ± 8.34	43	33.68 ± 11.46	0.71	0.08
	T1	57	32.25 ± 8.34	61	33.59 ± 10.12	0.43	-0.14

	T2	57	31.98 ± 7.89	61	31.94 ± 10.01	0.98	0.00
	T1-T0	41	-1.63 ± 3.53	42	0.00 ± 3.35	0.034	-0.47
	T2-T0	43	-2.50 ± 3.72	43	-1.55 ± 3.56	0.23	-0.26
	T2-T1	55	-0.53 ± 2.76	60	-1.43 ± 3.64	0.14	0.28
Inflammatory cytokine							
TNFα (pg/ml)	T0	59	579.58 ± 203.11	60	570.66 ± 181.18	0.8	0.05
	T1	57	580.05 ± 155.51	61	605.66 ± 136.50	0.35	-0.18
	T2	57	562.52 ± 161.12	61	595.16 ± 192.40	0.32	-0.18
	T1-T0	57	10.93 ± 251.70	59	23.65 ± 196.13	0.76	-0.06
	T2-T0	57	-15.79 ± 270.19	59	17.89 ± 233.43	0.47	-0.13
	T2-T1	55	-9.15 ± 175.69	60	-4.05 ± 152.06	0.87	-0.03
IL6 (pg/ml)	T0	57	30.61 ± 13.81	59	30.46 ± 13.06	0.95	0.01
	T1	57	16.51 ± 5.85	61	17.51 ± 5.54	0.34	-0.18
	T2	57	28.62 ± 13.79	60	30.41 ± 15.36	0.51	-0.12
	T1-T0	55	-12.85 ± 10.13	58	-12.74 ± 12.66	0.96	-0.01
	T2-T0	55	-1.74 ± 14.51	57	-0.13 ± 15.16	0.57	-0.11
	T2-T1	55	12.11 ± 11.87	59	13.26 ± 12.63	0.62	-0.09
Oxidative stress and Gene stability							
MDA (nmol/ml)	T0	59	2.11 ± 0.93	62	1.75 ± 0.71	0.02	0.43
	T1	53	3.74 ± 6.30	57	4.07 ± 6.57	0.79	-0.05
	T2	57	22.33 ± 27.57	61	18.26 ± 21.85	0.38	0.16
	T1-T0	53	1.61 ± 6.33	57	2.35 ± 6.70	0.56	-0.11
	T2-T0	57	20.19 ± 27.17	61	16.52 ± 21.62	0.42	0.15
	T2-T1	53	19.57 ± 28.63	56	14.55 ± 22.59	0.31	0.20
	T0	59	691.78 ± 232.93	62	660.32 ± 201.45	0.43	0.14

γH2AX (pg/ml)	T1	53	634.25 ± 282.65	57	655.76 ± 332.11	0.71	-0.07
	T2	57	687.03 ± 237.04	61	706.31 ± 240.29	0.66	-0.08
	T1-T0	53	-65.14 ± 312.29	57	-2.23 ± 413.06	0.37	-0.17
	T2-T0	57	-10.06 ± 313.68	61	44.19 ± 281.67	0.33	-0.18
	T2-T1	53	41.00 ± 362.56	56	50.90 ± 435.85	0.9	-0.02

* **T0** refers to the baseline measurement, **T1** represents the midpoint measurement (11-week), and **T2** indicates the endpoint measurement (19-week). **T1-T0** represents the change from **T0** to **T1**, **T2-T0** represents the change from **T0** to **T2** and **T2-T1** represents the change from **T1** to **T2**.

* The ***P-values*** are derived from *t*-tests that compare the changes between Nucleotides group and Control group.

* ***Cohens'd*** is reported to quantify the effect size.

* Median DNAmAge, DNA methylation median age; HOMA-IR, Homeostasis Model Assessment of Insulin Resistance; FBG, Fasting Blood Glucose; INS, Insulin; HbA1c, Hemoglobin A1c; LDL-C, Lipoprotein Cholesterol; TC, Total Cholesterol; TG, Triglycerides; TNFα, Tumor Necrosis Factor α; IL6, Interleukin-6; MDA, Malondialdehyde; γH2AX, γH2A Histone Family Member X.

Table S4. Generalized Estimating Equations of Primary and Secondary outcomes include baseline and 19-week.

Variable	Time	Mean difference in change between group (Nucleotides Group vs Control group)								
		Model 1			Model 2			Model 3		
		β	95%CI	*P*	β	95%CI	*P*	β	95%CI	*P*
Primary outcomes										
Leukocyte Telomere length (T/S ratio)	T2vsT0	0.11	(-0.06, 0.28)	0.19	0.11	(-0.06, 0.28)	0.2	0.11	(-0.06, 0.28)	0.19
Median DNAmAge (years)	T2vsT0	**-3.08**	**(-5.07, -1.10)**	**0.0023**	**-3.08**	**(-5.07, -1.09)**	**0.0024**	**-3.08**	**(-5.07, -1.09)**	**0.0024**
Glycolipid metabolic profile										
HOMA-IR	T2vsT0	**-0.45**	**(-0.86, -0.04)**	**0.033**	**-0.45**	**(-0.86, -0.03)**	**0.034**	**-0.45**	**(-0.86, -0.04)**	**0.033**
FBG (mmol/L)	T2vsT0	-0.08	(-0.37, 0.20)	0.56	-0.08	(-0.37, 0.21)	0.58	-0.09	(-0.37, 0.20)	0.56
INS (uIU/ml)	T2vsT0	**-1.28**	**(-2.30, -0.26)**	**0.014**	**-1.27**	**(-2.29, -0.25)**	**0.014**	**-1.27**	**(-2.29, -0.25)**	**0.014**
HBA1c (%)	T2vsT0	0.06	(-0.04, 0.17)	0.24	0.06	(-0.04, 0.17)	0.23	0.06	(-0.04, 0.17)	0.24
LDL-C (mmol/L)	T2vsT0	-0.05	(-0.23, 0.13)	0.61	-0.05	(-0.23, 0.13)	0.6	-0.05	(-0.23, 0.13)	0.6
TC (mmol/L)	T2vsT0	-0.07	(-0.34, 0.20)	0.62	-0.07	(-0.34, 0.20)	0.62	-0.07	(-0.34, 0.20)	0.62
TG (mmol/L)	T2vsT0	0.06	(-0.22, 0.34)	0.67	0.06	(-0.22, 0.34)	0.67	0.06	(-0.22, 0.34)	0.67
T lymphocyte subsets										
CD4+/CD8+ ratio	T2vsT0	0.14	(-0.01, 0.30)	0.061	0.15	(-0.01, 0.30)	0.06	0.15	(-0.01, 0.30)	0.06
CD3+CD4+ (%)	T2vsT0	1.59	(-0.28, 3.46)	0.095	1.61	(-0.26, 3.47)	0.092	1.60	(-0.27, 3.46)	0.094
CD3+CD8+ (%)	T2vsT0	-0.95	(-2.48, 0.57)	0.22	-0.95	(-2.48, 0.57)	0.22	-0.96	(-2.48, 0.57)	0.22
Inflammatory cytokine										
TNFα (pg/ml)	T2vsT0	-41.37	(-131.31, 48.57)	0.37	-41.48	(-131.19, 48.23)	0.36	-41.50	(-131.34, 48.34)	0.37
IL6 (pg/ml)	T2vsT0	-1.75	(-7.08, 3.58)	0.52	-1.72	(-7.06, 3.61)	0.53	-1.76	(-7.09, 3.58)	0.52
Oxidative stress and Gene stability										

MDA (nmol/ml)	T2vsT0	3.70	(-5.13, 12.53)	0.41	3.82	(-5.02, 12.66)	0.4	3.69	(-5.14, 12.52)	0.41
γH2AX (pg/ml)	T2vsT0	-51.73	(-157.76, 54.31)	0.34	-51.26	(-157.50, 54.98)	0.34	-52.04	(-158.06, 53.97)	0.34

* **T0** refers to the baseline measurement, **T2** indicates the endpoint measurement (19-week), **T2vsT0** represents the change from T0 to T2.

* ***P-values*** are based on repeated measures analysis with the interaction between the assignment group and time as well as covariates in the model.

* Primary assessment timepoints include baseline and 19-week.

* GEE **Model 1**: Adjusted covariates were baseline values of the measures, age, sex;
  GEE **Model 2**: Adjusted covariates were baseline values of the measures, age, sex and changes in dietary nucleotides intake;
  GEE **Model 3**: Adjusted covariates were baseline values of the measures, age, sex and changes in dietary purine intake.

* Bolded entries fall below the significance threshold of ***P*< 0.05**, while primary outcomes are based on a significance threshold of ***P*< 0.025**.

* Median DNAmAge, DNA methylation median age; HOMA-IR, Homeostasis Model Assessment of Insulin Resistance; FBG, Fasting Blood Glucose; INS, Insulin; HbA1c, Hemoglobin A1c; LDL-C, Lipoprotein Cholesterol; TC, Total Cholesterol; TG, Triglycerides; TNFα, Tumor Necrosis Factor α; IL6, Interleukin-6; MDA, Malondialdehyde; γH2AX, γH2A Histone Family Member X.

Table S5. Sensitivity analysis of Primary and Secondary outcomes include baseline, 11-week, and 19-week.

Variable	Time	Mean difference in change between group (Nucleotides group vs Control group)								
		Model 1			Model 2			Model 3		
		**β**	**95%CI**	***P***	**β**	**95%CI**	***P***	**β**	**95%CI**	***P***
Primary outcomes										
Leukocyte Telomere length (T/S ratio)	T1vsT0	0.09	(-0.10, 0.29)	0.36	0.09	(-0.10, 0.29)	0.36	0.09	(-0.10, 0.29)	0.36
	T2vsT0	0.12	(-0.05, 0.28)	0.18	0.11	(-0.05, 0.28)	0.18	0.12	(-0.05, 0.28)	0.18
Median DNAmAge (years)	T1vsT0	-1.94	(-4.32, 0.45)	0.11	-1.93	(-4.32, 0.45)	0.11	-1.94	(-4.32, 0.44)	0.11
	T2vsT0	**-3.08**	**(-5.07, -1.09)**	**0.0024**	**-3.07**	**(-5.06, -1.09)**	**0.0024**	**-3.08**	**(-5.06, -1.09)**	**0.0024**
Glycolipid metabolic profile										
HOMA-IR	T1vsT0	**-0.41**	**(-0.82, -0.01)**	**0.046**	**-0.41**	**(-0.82, -0.01)**	**0.046**	**-0.41**	**(-0.81, -0.01)**	**0.046**
	T2vsT0	**-0.45**	**(-0.86, -0.04)**	**0.032**	**-0.45**	**(-0.86, -0.04)**	**0.033**	**-0.45**	**(-0.86, -0.04)**	**0.032**
FBG (mmol/L)	T1vsT0	**-0.33**	**(-0.59, -0.06)**	**0.017**	**-0.33**	**(-0.59, -0.06)**	**0.017**	**-0.33**	**(-0.59, -0.06)**	**0.017**
	T2vsT0	-0.09	(-0.37, 0.20)	0.56	-0.08	(-0.37, 0.20)	0.56	-0.09	(-0.37, 0.20)	0.56
INS (uIU/ml)	T1vsT0	-0.83	(-1.96, 0.30)	0.15	-0.83	(-1.96, 0.30)	0.15	-0.83	(-1.96, 0.30)	0.15
	T2vsT0	**-1.27**	**(-2.28, -0.25)**	**0.014**	**-1.27**	**(-2.28, -0.25)**	**0.014**	**-1.27**	**(-2.28, -0.25)**	**0.014**
HBA1c (%)	T1vsT0	0.04	(-0.07, 0.15)	0.51	0.04	(-0.07, 0.15)	0.51	0.04	(-0.07, 0.15)	0.51
	T2vsT0	0.06	(-0.04, 0.17)	0.24	0.06	(-0.04, 0.17)	0.23	0.06	(-0.04, 0.17)	0.24
LDL-C (mmol/L)	T1vsT0	-0.03	(-0.23, 0.18)	0.78	-0.03	(-0.23, 0.18)	0.78	-0.03	(-0.23, 0.18)	0.78
	T2vsT0	-0.04	(-0.22, 0.13)	0.63	-0.04	(-0.22, 0.13)	0.62	-0.04	(-0.22, 0.13)	0.62
TC (mmol/L)	T1vsT0	-0.17	(-0.43, 0.08)	0.18	-0.17	(-0.43, 0.08)	0.18	-0.17	(-0.43, 0.08)	0.18
	T2vsT0	-0.06	(-0.33, 0.21)	0.64	-0.06	(-0.33, 0.21)	0.64	-0.06	(-0.33, 0.21)	0.64
TG (mmol/L)	T1vsT0	-0.04	(-0.27, 0.19)	0.72	-0.04	(-0.27, 0.19)	0.72	-0.04	(-0.27, 0.19)	0.72
	T2vsT0	0.06	(-0.22, 0.34)	0.68	0.06	(-0.22, 0.33)	0.68	0.06	(-0.22, 0.34)	0.68
T lymphocyte subsets										

CD4+/CD8+ ratio	T1vsT0	**0.22**	**(0.06, 0.37)**	**0.0052**	**0.22**	**(0.06, 0.37)**	**0.0052**	**0.22**	**(0.06, 0.37)**	**0.0051**
	T2vsT0	0.13	(-0.02, 0.28)	0.088	0.13	(-0.02, 0.28)	0.088	0.13	(-0.02, 0.28)	0.088
CD3+CD4+ (%)	T1vsT0	1.59	(-0.14, 3.32)	0.072	1.59	(-0.15, 3.32)	0.073	1.59	(-0.14, 3.32)	0.072
	T2vsT0	1.56	(-0.25, 3.38)	0.091	1.57	(-0.24, 3.38)	0.09	1.57	(-0.24, 3.38)	0.09
CD3+CD8+ (%)	T1vsT0	**-1.76**	**(-3.17, -0.35)**	**0.014**	**-1.76**	**(-3.17, -0.35)**	**0.014**	**-1.76**	**(-3.17, -0.36)**	**0.014**
	T2vsT0	-0.83	(-2.30, 0.63)	0.26	-0.84	(-2.30, 0.63)	0.26	-0.84	(-2.30, 0.62)	0.26
Inflammatory cytokine										
TNFα (pg/ml)	T1vsT0	-29.84	(-111.67, 51.98)	0.47	-29.85	(-111.68, 51.97)	0.47	-29.83	(-111.65, 51.99)	0.47
	T2vsT0	-38.60	(-128.45, 51.24)	0.4	-38.66	(-128.43, 51.11)	0.4	-38.59	(-128.38, 51.20)	0.4
IL6 (pg/ml)	T1vsT0	-0.71	(-4.97, 3.55)	0.74	-0.71	(-4.97, 3.55)	0.74	-0.71	(-4.97, 3.55)	0.74
	T2vsT0	-1.80	(-7.11, 3.51)	0.51	-1.79	(-7.09, 3.52)	0.51	-1.80	(-7.11, 3.50)	0.51
Oxidative stress and Gene stability										
MDA (nmol/ml)	T1vsT0	-0.72	(-3.15, 1.71)	0.56	-0.58	(-3.07, 1.91)	0.65	-0.68	(-3.13, 1.77)	0.59
	T2vsT0	3.71	(-5.12, 12.53)	0.41	3.79	(-5.05, 12.62)	0.4	3.70	(-5.13, 12.52)	0.41
γH2AX (pg/ml)	T1vsT0	-52.67	(-185.82, 80.49)	0.44	-51.27	(-184.06, 81.51)	0.45	-51.45	(-184.28, 81.37)	0.45
	T2vsT0	-51.19	(-157.18, 54.80)	0.34	-50.37	(-156.59, 55.85)	0.35	-51.47	(-157.47, 54.53)	0.34

* **T0** refers to the baseline measurement, **T1** represents the midpoint measurement (11-week), and **T2** indicates the endpoint measurement (19-week). **T1vsT0** represents the change from T0 to T1, **T2vsT0** represents the change from T0 to T2.
* ***P-values*** are based on repeated measures analysis with the interaction between the assignment group and time as well as covariates in the model.
* Primary assessment timepoints include baseline and 19-week.
* GEE **Model 1**: Adjusted covariates were baseline values of the measures, age, sex;
GEE **Model 2**: Adjusted covariates were baseline values of the measures, age, sex and changes in dietary nucleotides intake;
GEE **Model 3**: Adjusted covariates were baseline values of the measures, age, sex and changes in dietary purine intake.
* Bolded entries fall below the significance threshold of ***P*< 0.05**, while primary outcomes are based on a significance threshold of ***P*< 0.025**.

* Median DNAmAge, DNA methylation median age; HOMA-IR, Homeostasis Model Assessment of Insulin Resistance; FBG, Fasting Blood Glucose; INS, Insulin; HbA1c, Hemoglobin A1c; LDL-C, Lipoprotein Cholesterol; TC, Total Cholesterol; TG, Triglycerides; TNFα, Tumor Necrosis Factor α; IL6, Interleukin-6; MDA, Malondialdehyde; γH2AX, γH2A Histone Family Member X.

Table S6. Other outcomes for TALENTs participants in the Nucleotides group and Control group at baseline, 11-week, and 19-week assessments.

Variable	Time	Nucleotides group (n=59)		Control group (n=62)		*P*	Cohen's *d*
		N	Mean±SD	N	Mean±SD		
Body composition							
Skeletal muscle mass (kg)	T0	58	39.16 ± 7.27	62	40.29 ± 6.40	0.37	-0.17
	T1	40	38.90 ± 7.07	41	39.91 ± 6.58	0.51	-0.15
	T2	57	39.47 ± 7.41	61	40.19 ± 6.40	0.57	-0.10
	T1-T0	39	0.21 ± 1.52	41	0.04 ± 0.63	0.53	0.14
	T2-T0	56	0.36 ± 1.69	61	-0.13 ± 0.93	0.056	0.37
	T2-T1	38	0.14 ± 1.09	40	-0.27 ± 0.83	0.067	0.42
Trunk muscle mass (kg)	T0	58	21.90 ± 3.77	62	22.49 ± 3.32	0.36	-0.17
	T1	40	21.71 ± 3.63	41	22.29 ± 3.38	0.46	-0.17
	T2	57	22.01 ± 3.86	61	22.43 ± 3.28	0.53	-0.12
	T1-T0	39	0.03 ± 0.47	41	0.04 ± 0.33	0.95	-0.01
	T2-T0	56	0.13 ± 0.76	61	-0.08 ± 0.53	0.085	0.33
	T2-T1	38	0.06 ± 0.68	40	-0.15 ± 0.47	0.11	0.37
Appendicular lean mass (kg)	T0	58	17.38 ± 3.52	62	17.76 ± 3.08	0.53	-0.12
	T1	40	17.80 ± 5.76	41	17.63 ± 3.20	0.87	0.04
	T2	57	17.45 ± 3.58	61	17.78 ± 3.12	0.61	-0.10
	T1-T0	39	0.64 ± 3.79	41	0.06 ± 0.27	0.35	0.22
	T2-T0	56	0.10 ± 0.55	61	-0.01 ± 0.45	0.25	0.22
	T2-T1	38	-0.57 ± 3.90	40	-0.11 ± 0.37	0.48	-0.17
Body fat rate (%)	T0	58	29.63 ± 6.46	62	30.21 ± 7.94	0.66	-0.08
	T1	40	29.07 ± 6.72	41	29.90 ± 8.01	0.61	-0.11

	T2	57	29.83 ± 6.91	61	30.76 ± 7.85	0.49	-0.13
	T1-T0	39	-0.29 ± 1.96	41	-0.43 ± 1.28	0.71	0.08
	T2-T0	56	-0.13 ± 2.41	61	0.70 ± 2.04	0.047	-0.37
	T2-T1	38	0.47 ± 2.02	40	0.94 ± 2.00	0.3	-0.24
Fat mass (kg)	T0	58	17.73 ± 5.18	62	18.96 ± 6.84	0.27	-0.20
	T1	40	17.13 ± 5.26	41	18.49 ± 6.95	0.32	-0.22
	T2	57	18.11 ± 5.93	61	19.39 ± 6.91	0.28	-0.20
	T1-T0	39	-0.20 ± 1.36	41	-0.39 ± 1.16	0.51	0.15
	T2-T0	56	0.05 ± 1.55	61	0.56 ± 1.49	0.074	-0.33
	T2-T1	38	0.46 ± 1.19	40	0.74 ± 1.33	0.33	-0.22
Trunk Fat mass (kg)	T0	58	8.94 ± 2.80	62	9.55 ± 3.71	0.31	-0.19
	T1	40	8.59 ± 2.86	41	9.31 ± 3.78	0.34	-0.21
	T2	57	9.13 ± 3.21	61	9.83 ± 3.73	0.27	-0.20
	T1-T0	39	-0.11 ± 0.77	41	-0.20 ± 0.68	0.62	0.11
	T2-T0	56	0.01 ± 0.89	61	0.35 ± 0.86	0.04	-0.38
	T2-T1	38	0.24 ± 0.69	40	0.42 ± 0.76	0.27	-0.25
Visceral fat grade	T0	58	13.52 ± 2.47	62	13.71 ± 3.20	0.71	-0.07
	T1	40	13.32 ± 2.56	41	13.66 ± 3.15	0.6	-0.12
	T2	57	13.58 ± 2.56	61	14.02 ± 3.12	0.4	-0.15
	T1-T0	39	-0.13 ± 0.92	41	-0.12 ± 0.60	0.97	-0.01
	T2-T0	56	-0.05 ± 0.96	61	0.36 ± 0.84	0.015	-0.46
	T2-T1	38	0.24 ± 0.82	40	0.38 ± 0.77	0.45	-0.17
Comprehensive Geriatric Assessment-Questionnaire							
Frailty index	T0	59	0.11 ± 0.06	62	0.10 ± 0.06	0.53	0.12
	T2	57	0.13 ± 0.08	61	0.13 ± 0.08	0.84	-0.04

	T2-T0	57	0.02 ± 0.07	61	0.03 ± 0.07	0.49	-0.13
Short Form-12 Health Survey	T0	59	107.51 ± 7.31	62	108.42 ± 7.83	0.51	-0.12
	T2	57	108.05 ± 9.77	61	107.91 ± 9.42	0.94	0.01
	T2-T0	57	0.81 ± 9.26	61	-0.46 ± 10.10	0.48	0.13
Fatigue Scale-14	T0	59	4.98 ± 3.55	62	4.21 ± 3.24	0.21	0.23
	T2	57	3.42 ± 3.01	61	4.03 ± 3.34	0.3	-0.19
	T2-T0	57	-1.65 ± 3.03	61	-0.23 ± 3.31	0.017	-0.45
Physical Fatigue	T0	59	51.85 ± 3.94	62	52.58 ± 4.78	0.36	-0.17
	T2	57	50.65 ± 6.12	61	50.95 ± 5.59	0.78	-0.05
	T2-T0	57	-1.11 ± 6.20	61	-1.59 ± 6.45	0.68	0.08
Mental fatigue	T0	59	55.65 ± 6.06	62	55.84 ± 6.62	0.87	-0.03
	T2	57	57.40 ± 6.22	61	56.96 ± 7.01	0.72	0.07
	T2-T0	57	1.92 ± 7.23	61	1.13 ± 8.27	0.58	0.10
Montreal Cognitive Assessment	T0	59	21.46 ± 3.89	62	22.32 ± 3.84	0.22	-0.22
	T2	57	22.44 ± 3.45	61	22.57 ± 3.60	0.84	-0.04
	T2-T0	57	1.09 ± 2.81	61	0.18 ± 3.32	0.11	0.29
Pittsburgh Sleep Quality Index	T0	59	4.51 ± 3.30	62	5.21 ± 3.85	0.28	-0.20
	T2	57	5.75 ± 3.70	61	5.41 ± 3.96	0.63	0.09
	T2-T0	57	1.30 ± 4.85	61	0.21 ± 5.06	0.24	0.22
Kessler 10 scale	T0	59	11.29 ± 1.96	62	11.84 ± 3.37	0.27	-0.20
	T2	57	10.75 ± 1.73	61	11.48 ± 3.36	0.14	-0.27
	T2-T0	57	-0.58 ± 2.84	61	-0.39 ± 4.99	0.8	-0.05

* **T0** refers to the baseline measurement, **T1** represents the midpoint measurement (11-week), and **T2** indicates the endpoint measurement (19-week). **T1-T0** represents the change from **T0** to **T1**, **T2-T0** represents the change from **T0** to **T2** and **T2-T1** represents the change from **T1** to **T2**.

* The ***P-values*** are derived from *t*-tests that compare the changes between Nucleotides group and Control group.
* ***Cohens'd*** is reported to quantify the effect size.

Table S7. Generalized Estimating Equations of Other outcomes include baseline and 19-week.

Variable	Time	Mean difference in change between group (Nucleotides group vs Control group)								
		Model 1			Model 2			Model 3		
		**β**	**95%CI**	***P***	**β**	**95%CI**	***P***	**β**	**95%CI**	***P***
Body composition										
Skeletal muscle mass (kg)	T2vsT0	**0.50**	**(0.01, 1.00)**	**0.046**	**0.50**	**(0.01, 1.00)**	**0.046**	**0.50**	**(0.01, 1.00)**	**0.046**
Trunk muscle mass (kg)	T2vsT0	0.22	(-0.02, 0.45)	0.073	0.22	(-0.02, 0.45)	0.072	0.22	(-0.02, 0.45)	0.072
Appendicular lean mass (kg)	T2vsT0	0.11	(-0.07, 0.29)	0.23	0.11	(-0.07, 0.29)	0.23	0.11	(-0.07, 0.29)	0.23
Body fat rate (%)	T2vsT0	**-0.80**	**(-1.61, 0.00)**	**0.05**	-0.80	(-1.61, 0.01)	0.052	**-0.80**	**(-1.61, 0.00)**	**0.05**
Fat mass (kg)	T2vsT0	-0.49	(-1.04, 0.06)	0.082	-0.48	(-1.03, 0.06)	0.084	-0.49	(-1.04, 0.06)	0.082
Trunk Fat mass (kg)	T2vsT0	**-0.33**	**(-0.64, -0.01)**	**0.044**	**-0.32**	**(-0.64, -0.01)**	**0.046**	**-0.33**	**(-0.64, -0.01)**	**0.044**
Visceral fat grade	T2vsT0	**-0.40**	**(-0.73, -0.08)**	**0.015**	**-0.40**	**(-0.73, -0.08)**	**0.015**	**-0.40**	**(-0.73, -0.08)**	**0.015**
Comprehensive Geriatric Assessment-Questionnaire										
Frailty index	T2vsT0	-0.01	(-0.04, 0.02)	0.46	-0.01	(-0.03, 0.02)	0.47	-0.01	(-0.04, 0.02)	0.46
Short Form-12 Health Survey	T2vsT0	1.16	(-2.30, 4.62)	0.51	1.16	(-2.29, 4.62)	0.51	1.15	(-2.30, 4.61)	0.51
Fatigue Scale-14	T2vsT0	**-1.41**	**(-2.54, -0.28)**	**0.014**	**-1.40**	**(-2.53, -0.27)**	**0.015**	**-1.41**	**(-2.54, -0.28)**	**0.014**
Physical Fatigue	T2vsT0	0.46	(-1.79, 2.71)	0.69	0.46	(-1.80, 2.71)	0.69	0.45	(-1.80, 2.70)	0.7
Mental fatigue	T2vsT0	0.69	(-2.07, 3.45)	0.63	0.69	(-2.06, 3.45)	0.62	0.69	(-2.06, 3.45)	0.62
Montreal Cognitive Assessment	T2vsT0	0.85	(-0.25, 1.94)	0.13	0.84	(-0.26, 1.93)	0.14	0.85	(-0.25, 1.94)	0.13
Pittsburgh Sleep Quality Index	T2vsT0	1.04	(-0.72, 2.80)	0.25	1.04	(-0.73, 2.80)	0.25	1.03	(-0.73, 2.80)	0.25
Kessler 10 scale	T2vsT0	-0.18	(-1.60, 1.25)	0.81	-0.17	(-1.59, 1.25)	0.82	-0.18	(-1.61, 1.25)	0.8

* **T0** refers to the baseline measurement, **T2** indicates the endpoint measurement (19-week), and **T2vsT0** represents the change from T0 to T2.

* ***P-values*** are based on repeated measures analysis with the interaction between the assignment group and time as well as covariates in the model.

* Primary assessment timepoints include baseline and 19-week.

* GEE **Model 1**: Adjusted covariates were baseline values of the measures, age, sex;
GEE **Model 2**: Adjusted covariates were baseline values of the measures, age, sex and changes in dietary nucleotides intake;
GEE **Model 3**: Adjusted covariates were baseline values of the measures, age, sex and changes in dietary purine intake.
* Bolded entries fall below the significance threshold of ***P*< 0.05**.

Table S8. Safety indicators of Tumor biomarker, Routine examination of blood, Liver and kidney function for TALENTs participants in the Nucleotides group and Control group at baseline, 11-week, and 19-week assessments.

Variable	Time	Nucleotides group (n=59)		Control group (n=62)		*P*	Cohen's *d*
		N	Mean±SD	N	Mean±SD		
Tumor biomarker							
CEA (ng/ml)	T0	59	2.36 ± 0.69	62	2.27 ± 0.59	0.45	0.14
	T1	57	2.51 ± 0.77	61	2.46 ± 0.76	0.68	0.08
	T2	57	2.78 ± 0.84	61	2.76 ± 0.92	0.93	0.02
	T1-T0	57	0.14 ± 0.59	61	0.18 ± 0.69	0.72	-0.07
	T2-T0	57	0.42 ± 0.56	61	0.50 ± 0.71	0.53	-0.11
	T2-T1	55	0.26 ± 0.55	60	0.31 ± 0.66	0.69	-0.07
AFP (ng/ml)	T0	59	6.73 ± 2.02	62	7.58 ± 2.36	0.034	-0.39
	T1	57	6.36 ± 1.88	61	6.84 ± 2.01	0.19	-0.24
	T2	57	7.68 ± 1.79	61	8.08 ± 2.17	0.27	-0.20
	T1-T0	57	-0.32 ± 1.77	61	-0.77 ± 2.20	0.22	0.23
	T2-T0	57	0.92 ± 1.75	61	0.52 ± 1.86	0.24	0.22
	T2-T1	55	1.30 ± 1.88	60	1.27 ± 1.85	0.93	0.02
Liver and kidney function							
ALT (U/L)	T0	59	24.49 ± 10.52	62	26.27 ± 14.93	0.45	-0.14
	T1	57	20.53 ± 10.10	61	23.07 ± 10.62	0.19	-0.24
	T2	57	21.70 ± 11.95	61	24.05 ± 14.72	0.34	-0.17
	T1-T0	57	-2.96 ± 9.26	61	-3.44 ± 11.66	0.81	0.05
	T2-T0	57	-3.09 ± 11.52	61	-2.31 ± 13.59	0.74	-0.06
	T2-T1	55	0.11 ± 13.23	60	0.95 ± 14.66	0.75	-0.06
AST (U/L)	T0	59	24.83 ± 7.06	62	24.79 ± 8.30	0.98	0.01

	T1	57	22.56 ± 5.17	61	23.54 ± 8.55	0.45	-0.14
	T2	57	23.86 ± 5.90	61	24.34 ± 7.75	0.7	-0.07
	T1-T0	57	-1.56 ± 5.75	61	-1.39 ± 9.32	0.91	-0.02
	T2-T0	57	-1.07 ± 5.58	61	-0.56 ± 7.09	0.66	-0.08
	T2-T1	55	0.58 ± 5.33	60	0.78 ± 9.34	0.89	-0.03
ALP (U/L)	T0	59	84.86 ± 24.73	62	85.85 ± 29.65	0.84	-0.04
	T1	57	82.56 ± 23.98	61	83.30 ± 29.70	0.88	-0.03
	T2	57	80.21 ± 25.33	61	81.30 ± 28.66	0.83	-0.04
	T1-T0	57	-2.26 ± 12.24	61	-2.95 ± 13.36	0.77	0.05
	T2-T0	57	-5.65 ± 11.14	61	-4.57 ± 16.46	0.68	-0.08
	T2-T1	55	-2.98 ± 8.62	60	-1.52 ± 11.41	0.44	-0.14
TBA (umol/L)	T0	59	2.71 ± 2.30	62	3.00 ± 3.79	0.61	-0.09
	T1	57	4.60 ± 2.68	61	4.21 ± 3.60	0.51	0.12
	T2	57	4.57 ± 3.93	61	4.58 ± 2.71	0.99	-0.00
	T1-T0	57	1.88 ± 2.99	61	1.21 ± 2.68	0.2	0.24
	T2-T0	57	1.85 ± 4.18	61	1.56 ± 3.01	0.67	0.08
	T2-T1	55	0.10 ± 3.91	60	0.31 ± 2.86	0.75	-0.06
Cys-C (mg/L)	T0	59	1.10 ± 0.22	62	1.09 ± 0.18	0.71	0.07
	T1	57	1.22 ± 0.21	61	1.25 ± 0.21	0.34	-0.18
	T2	57	1.09 ± 0.16	61	1.10 ± 0.15	0.6	-0.10
	T1-T0	57	0.13 ± 0.21	61	0.17 ± 0.24	0.34	-0.18
	T2-T0	57	-0.02 ± 0.14	61	0.01 ± 0.17	0.27	-0.20
	T2-T1	55	-0.14 ± 0.15	60	-0.15 ± 0.16	0.64	0.09
UA (umol/L)	T0	59	295.58 ± 70.30	62	284.58 ± 57.30	0.35	0.17
	T1	57	317.49 ± 70.30	61	276.59 ± 70.22	0.002	0.58

	T2	57	321.93 ± 72.86	61	298.52 ± 63.22	0.066	0.34
	T1-T0	57	25.02 ± 42.35	61	-7.31 ± 44.49	0.000095	0.74
	T2-T0	57	29.11 ± 53.91	61	13.57 ± 40.92	0.082	0.33
	T2-T1	55	3.16 ± 48.78	60	20.65 ± 39.39	0.038	-0.40
β2-MG (μg/L)	T0	59	1617.95 ± 381.85	62	1566.88 ± 280.40	0.41	0.15
	T1	57	2201.16 ± 381.00	61	2222.20 ± 414.01	0.77	-0.05
	T2	57	1662.06 ± 432.84	61	1613.92 ± 322.11	0.5	0.13
	T1-T0	57	623.14 ± 256.77	61	657.20 ± 246.52	0.46	-0.14
	T2-T0	57	42.33 ± 382.09	61	47.88 ± 216.98	0.92	-0.02
	T2-T1	55	-564.18 ± 414.01	60	-601.39 ± 273.91	0.57	0.11
U-ALB (g/L)	T0	59	13.40 ± 11.72	62	13.74 ± 11.30	0.87	-0.03
	T1	57	16.76 ± 10.31	61	24.19 ± 30.38	0.075	-0.32
	T2	57	16.02 ± 11.13	61	18.88 ± 20.65	0.35	-0.17
	T1-T0	57	3.67 ± 9.13	61	10.35 ± 28.88	0.09	-0.31
	T2-T0	57	2.99 ± 11.38	61	5.04 ± 14.79	0.4	-0.16
	T2-T1	55	-1.50 ± 8.63	60	-5.26 ± 28.17	0.33	0.18
Routine examination of blood							
RBC (10^9/L)	T0	59	4.60 ± 0.52	62	4.48 ± 0.44	0.17	0.25
	T1	57	4.42 ± 0.45	61	4.38 ± 0.38	0.58	0.10
	T2	57	4.37 ± 0.45	61	4.31 ± 0.38	0.45	0.14
	T1-T0	57	-0.17 ± 0.23	61	-0.10 ± 0.26	0.11	-0.30
	T2-T0	57	-0.22 ± 0.19	61	-0.18 ± 0.28	0.33	-0.18
	T2-T1	55	-0.05 ± 0.18	60	-0.07 ± 0.22	0.58	0.10
WBC (10^9/L)	T0	59	5.78 ± 1.57	62	5.57 ± 1.20	0.41	0.15
	T1	57	5.54 ± 1.22	61	5.34 ± 1.12	0.38	0.16

	T2	57	5.31 ± 1.33	61	5.18 ± 1.08	0.56	0.11
	T1-T0	57	-0.26 ± 1.01	61	-0.21 ± 0.87	0.81	-0.04
	T2-T0	57	-0.51 ± 0.98	61	-0.39 ± 0.87	0.48	-0.13
	T2-T1	55	-0.25 ± 0.72	60	-0.17 ± 0.80	0.58	-0.10
Lymphocyte absolute value (10^9/L)	T0	59	1.87 ± 0.59	62	1.88 ± 0.51	0.86	-0.03
	T1	57	1.84 ± 0.62	61	1.86 ± 0.47	0.88	-0.03
	T2	57	1.77 ± 0.54	61	1.79 ± 0.44	0.85	-0.03
	T1-T0	57	-0.03 ± 0.44	61	-0.04 ± 0.44	0.96	0.01
	T2-T0	57	-0.11 ± 0.43	61	-0.08 ± 0.40	0.73	-0.06
	T2-T1	55	-0.08 ± 0.31	60	-0.06 ± 0.35	0.73	-0.06
Neutrophil absolute value (10^9/L)	T0	59	3.42 ± 1.27	62	3.21 ± 0.86	0.28	0.20
	T1	57	3.17 ± 0.79	61	3.00 ± 0.86	0.26	0.21
	T2	57	3.03 ± 0.97	61	2.90 ± 0.81	0.42	0.15
	T1-T0	57	-0.26 ± 0.95	61	-0.19 ± 0.63	0.64	-0.09
	T2-T0	57	-0.41 ± 1.01	61	-0.32 ± 0.62	0.56	-0.11
	T2-T1	55	-0.14 ± 0.65	60	-0.11 ± 0.61	0.78	-0.05

* **T0** refers to the baseline measurement, **T1** represents the midpoint measurement (11-week), and **T2** indicates the endpoint measurement (19-week). **T1-T0** represents the change from **T0** to **T1**, **T2-T0** represents the change from **T0** to **T2** and **T2-T1** represents the change from **T1** to **T2**.

* The ***P-values*** are derived from *t*-tests that compare the changes between Nucleotides group and Control group.

* ***Cohens'd*** is reported to quantify the effect size.

* CEA, Carcinoembryonic Antigen; AFP, Alpha-Fetoprotein; ALT, Alanine Aminotransferase; AST, Aspartate Aminotransferase; ALP, Alkaline Phosphatase; TBA, Total Bile Acids; Cys-C, Cystatin C; UA, Uric Acid; β2-MG, β-2 Microglobulin; U-ALB, Urinary Albumin; RBC, a Count; WBC, White Blood Cell Count.

Table S9. Generalized Estimating Equations of Safety indicators include baseline and 19-week

Variable	Time	Mean difference in change between group (Nucleotides group vs Control group)								
		Model 1			Model 2			Model 3		
		β	95%CI	*P*	β	95%CI	*P*	β	95%CI	*P*
Tumor biomarker										
CEA (ng/ml)	T2vsT0	-0.07	(-0.30, 0.15)	0.53	-0.07	(-0.30, 0.15)	0.53	-0.08	(-0.30, 0.15)	0.52
AFP (ng/ml)	T2vsT0	0.41	(-0.23, 1.05)	0.21	0.41	(-0.23, 1.06)	0.21	0.41	(-0.23, 1.05)	0.21
Liver and kidney function										
ALT (U/L)	T2vsT0	-0.68	(-5.16, 3.81)	0.77	-0.67	(-5.17, 3.83)	0.77	-0.66	(-5.15, 3.83)	0.77
AST (U/L)	T2vsT0	-0.51	(-2.78, 1.75)	0.66	-0.51	(-2.78, 1.75)	0.66	-0.51	(-2.77, 1.76)	0.66
ALP (U/L)	T2vsT0	-0.96	(-5.96, 4.04)	0.71	-0.98	(-5.98, 4.02)	0.7	-0.95	(-5.95, 4.04)	0.71
TBA (umol/L)	T2vsT0	0.29	(-1.02, 1.59)	0.67	0.28	(-1.02, 1.58)	0.68	0.29	(-1.02, 1.59)	0.67
Cys-C (mg/L)	T2vsT0	-0.03	(-0.09, 0.03)	0.30	-0.03	(-0.09, 0.03)	0.31	-0.03	(-0.09, 0.03)	0.31
UA (umol/L)	T2vsT0	14.74	(-2.45, 31.92)	0.093	14.63	(-2.56, 31.82)	0.095	14.75	(-2.45, 31.96)	0.093
β2-MG (μg/L)	T2vsT0	-4.20	(-116.02, 107.63)	0.94	-4.11	(-116.46, 108.25)	0.94	-3.91	(-116.00, 108.18)	0.95
U-ALB (g/L)	T2vsT0	-2.26	(-6.97, 2.46)	0.35	-2.24	(-6.95, 2.47)	0.35	-2.27	(-6.99, 2.45)	0.35
Routine examination of blood										
RBC (10^9/L)	T2vsT0	-0.05	(-0.13, 0.04)	0.29	-0.05	(-0.13, 0.04)	0.29	-0.05	(-0.13, 0.04)	0.29
WBC (10^9/L)	T2vsT0	-0.11	(-0.44, 0.22)	0.51	-0.11	(-0.44, 0.22)	0.52	-0.11	(-0.44, 0.22)	0.51
Lymphocyte absolute value (10^9/L)	T2vsT0	-0.02	(-0.17, 0.13)	0.81	-0.02	(-0.17, 0.13)	0.81	-0.02	(-0.17, 0.13)	0.81
Neutrophil absolute value (10^9/L)	T2vsT0	-0.09	(-0.39, 0.21)	0.57	-0.08	(-0.38, 0.22)	0.59	-0.09	(-0.39, 0.21)	0.57

* **T0** refers to the baseline measurement, **T2** indicates the endpoint measurement (19-week), **T2vsT0** represents the change from T0 to T2.

* ***P-values*** are based on repeated measures analysis with the interaction between the assignment group and time as well as covariates in the model.
* Primary assessment timepoints include baseline and 19-week.
* GEE **Model 1**: Adjusted covariates were baseline values of the measures, age, sex;
GEE **Model 2**: Adjusted covariates were baseline values of the measures, age, sex and changes in dietary nucleotides intake;
GEE **Model 3**: Adjusted covariates were baseline values of the measures, age, sex and changes in dietary purine intake.
* Bolded entries fall below the significance threshold of ***P*< 0.05**.
* CEA, Carcinoembryonic Antigen; AFP, Alpha-Fetoprotein; ALT, Alanine Aminotransferase; AST, Aspartate Aminotransferase; ALP, Alkaline Phosphatase; TBA, Total Bile Acids; Cys-C, Cystatin C; UA, Uric Acid; β2-MG, β-2 Microglobulin; U-ALB, Urinary Albumin; RBC, Red Blood Cell Count; WBC, White Blood Cell Count.

Table S10. Sensitivity analysis of Safety indicators include baseline, 11-week, and 19-week.

Variable	Time	Mean difference in change between group (Nucleotides group vs Control group)								
		Model 1			Model 2			Model 3		
		β	95%CI	*P*	β	95%CI	*P*	β	95%CI	*P*
Tumor biomarker										
CEA (ng/ml)	T1vsT0	-0.03	(-0.26, 0.19)	0.77	-0.03	(-0.26, 0.19)	0.77	-0.03	(-0.26, 0.19)	0.77
	T2vsT0	-0.07	(-0.30, 0.15)	0.53	-0.07	(-0.30, 0.15)	0.53	-0.07	(-0.30, 0.15)	0.52
AFP (ng/ml)	T1vsT0	0.43	(-0.28, 1.13)	0.24	0.43	(-0.28, 1.13)	0.24	0.43	(-0.28, 1.13)	0.24
	T2vsT0	0.44	(-0.21, 1.08)	0.18	0.44	(-0.21, 1.08)	0.18	0.43	(-0.21, 1.08)	0.18
Liver and kidney function										
ALT (U/L)	T1vsT0	-0.09	(-3.87, 3.69)	0.96	-0.09	(-3.87, 3.69)	0.96	-0.09	(-3.87, 3.70)	0.96
	T2vsT0	-0.70	(-5.17, 3.78)	0.76	-0.69	(-5.17, 3.79)	0.76	-0.68	(-5.16, 3.80)	0.76
AST (U/L)	T1vsT0	-0.53	(-3.30, 2.24)	0.71	-0.53	(-3.30, 2.24)	0.71	-0.53	(-3.30, 2.24)	0.71
	T2vsT0	-0.52	(-2.77, 1.73)	0.65	-0.52	(-2.77, 1.73)	0.65	-0.51	(-2.76, 1.74)	0.65
ALP (U/L)	T1vsT0	0.56	(-3.98, 5.10)	0.81	0.56	(-3.98, 5.10)	0.81	0.56	(-3.98, 5.10)	0.81
	T2vsT0	-0.99	(-5.95, 3.98)	0.70	-0.99	(-5.96, 3.97)	0.69	-0.99	(-5.95, 3.98)	0.70
TBA (umol/L)	T1vsT0	0.66	(-0.35, 1.67)	0.20	0.66	(-0.35, 1.67)	0.20	0.66	(-0.35, 1.67)	0.20
	T2vsT0	0.33	(-0.98, 1.64)	0.62	0.33	(-0.98, 1.63)	0.62	0.33	(-0.98, 1.63)	0.62
Cys-C (mg/L)	T1vsT0	-0.04	(-0.12, 0.04)	0.29	-0.04	(-0.12, 0.04)	0.29	-0.04	(-0.12, 0.04)	0.29
	T2vsT0	-0.03	(-0.09, 0.03)	0.32	-0.03	(-0.09, 0.03)	0.32	-0.03	(-0.09, 0.03)	0.32
UA (umol/L)	T1vsT0	**31.61**	**(16.15, 47.07)**	**0.000061**	**31.61**	**(16.15, 47.07)**	**0.000061**	**31.61**	**(16.15, 47.07)**	**0.000061**
	T2vsT0	14.39	(-2.79, 31.57)	0.10	14.35	(-2.83, 31.54)	0.10	14.39	(-2.80, 31.58)	0.10
β2-MG (μg/L)	T1vsT0	-50.38	(-145.71, 44.95)	0.30	-50.38	(-145.71, 44.95)	0.30	-50.36	(-145.70, 44.99)	0.30
	T2vsT0	-8.93	(-120.65, 102.80)	0.88	-9.01	(-121.01, 102.98)	0.87	-8.89	(-120.75, 102.97)	0.88
U-ALB (g/L)	T1vsT0	-6.68	(-14.22, 0.86)	0.083	-6.68	(-14.22, 0.86)	0.083	-6.69	(-14.24, 0.86)	0.082

	T2vsT0	-2.32	(-7.02, 2.38)	0.33	-2.30	(-6.99, 2.39)	0.34	-2.34	(-7.05, 2.37)	0.33
Routine examination of blood										
RBC (10^9/L)	T1vsT0	-0.07	(-0.16, 0.02)	0.10	-0.07	(-0.16, 0.02)	0.10	-0.07	(-0.16, 0.02)	0.10
	T2vsT0	-0.05	(-0.13, 0.04)	0.26	-0.05	(-0.13, 0.04)	0.26	-0.05	(-0.13, 0.04)	0.26
WBC (10^9/L)	T1vsT0	-0.04	(-0.37, 0.30)	0.84	-0.04	(-0.37, 0.30)	0.84	-0.04	(-0.37, 0.30)	0.84
	T2vsT0	-0.11	(-0.44, 0.22)	0.50	-0.11	(-0.44, 0.22)	0.51	-0.11	(-0.44, 0.22)	0.50
Lymphocyte absolute value (10^9/L)	T1vsT0	0.00	(-0.15, 0.16)	0.96	0.00	(-0.15, 0.16)	0.96	0.00	(-0.15, 0.16)	0.97
	T2vsT0	-0.02	(-0.17, 0.13)	0.81	-0.02	(-0.17, 0.13)	0.81	-0.02	(-0.17, 0.13)	0.81
Neutrophil absolute value (10^9/L)	T1vsT0	-0.06	(-0.35, 0.23)	0.68	-0.06	(-0.35, 0.23)	0.68	-0.06	(-0.35, 0.23)	0.69
	T2vsT0	-0.09	(-0.39, 0.21)	0.56	-0.09	(-0.39, 0.21)	0.57	-0.09	(-0.39, 0.21)	0.56

* **T0** refers to the baseline measurement, **T1** represents the midpoint measurement (11-week), and **T2** indicates the endpoint measurement (19-week). **T1vsT0** represents the change from T0 to T1, **T2vsT0** represents the change from T0 to T2.

* ***P-values*** are based on repeated measures analysis with the interaction between the assignment group and time as well as covariates in the model.

* Primary assessment timepoints include baseline and 19-week.

* GEE **Model 1**: Adjusted covariates were baseline values of the measures, age, sex;
GEE **Model 2**: Adjusted covariates were baseline values of the measures, age, sex and changes in dietary nucleotides intake;
GEE **Model 3**: Adjusted covariates were baseline values of the measures, age, sex and changes in dietary purine intake.

* Bolded entries fall below the significance threshold of ***P*< 0.05**.

* CEA, Carcinoembryonic Antigen; AFP, Alpha-Fetoprotein; ALT, Alanine Aminotransferase; AST, Aspartate Aminotransferase; ALP, Alkaline Phosphatase; TBA, Total Bile Acids; Cys-C, Cystatin C; UA, Uric Acid; β2-MG, β-2 Microglobulin; U-ALB, Urinary Albumin; RBC, Red Blood Cell Count; WBC, White Blood Cell Count.

Table S11. Adverse events for TALENTs participants.

	Overall (n=121)	Nucleotides group (n=59)	Control group (n=62)	*P*
**Adverse reaction symptoms (person)**				
Irritation (ulcers, puffiness)	3	3	0	0.11
Digestive discomfort	13	4	9	0.24
Dyssomnias	7	4	3	0.71
Suspected allergy	4	0	4	0.12
Others (unstable blood pressure, eye discomfort)	4	2	2	1.00
Total	31	14	17	0.68
**Time period of adverse reactions (person-time)**				
2022.10.23-11.31	20	8	12	0.47
2022.12.1-12.30	4	2	2	1.00
2023.1.1-1.31	5	4	1	0.20
2023.2.1-3.4	3	0	3	0.24
Total	32	14	18	0.54

* Multiple adverse reactions may occur in the same person, and the occurrence time of adverse reactions is recorded as the number of adverse reactions.

* The ***P-values*** are derived from Fisher's exact test comparing the number of adverse events between the Nucleotides and Control groups.

Table S12. Outcome measures assessed in the TALENTs study.

Category	Outcome（Units）	Source/Method	Baseline	Midpoint	Endpoint
**Primary Outcome**	Median DNAmAge（years）	Venous blood draw (fasted)	X	X	X
	Leukocyte Telomere length (T/S ratio)	Venous blood draw (fasted)	X	X	X
**Secondary Outcome**	**Glycolipid metabolic profile**				
	HOMA-IR	Venous blood draw (fasted)	X	X	X
	FBG (mmol/L)	Venous blood draw (fasted)	X	X	X
	INS (uIU/ml)	Venous blood draw (fasted)	X	X	X
	HBA1c (%)	Venous blood draw (fasted)	X	X	X
	LDL-C(mmol/L)	Venous blood draw (fasted)	X	X	X
	TC (mmol/L)	Venous blood draw (fasted)	X	X	X
	TG (mmol/L)	Venous blood draw (fasted)	X	X	X
	**T lymphocyte subsets**				
	CD4+/CD8+ ratio	Venous blood draw (fasted)	X	X	X
	CD3+CD4+（%）	Venous blood draw (fasted)	X	X	X
	CD3+CD8+（%）	Venous blood draw (fasted)	X	X	X
	**Inflammatory cytokine**				
	TNFα（pg/ml）	Venous blood draw (fasted)	X	X	X
	IL6（pg/ml）	Venous blood draw (fasted)	X	X	X
	**Oxidative stress and Gene stability**				
	MDA (nmol/ml)	Venous blood draw (fasted)	X	X	X
	γH2AX（pg/ml）	Venous blood draw (fasted)	X	X	X
**Other Outcome**	**Body composition**				
	Skeletal muscle mass（kg）	Bioelectrical Impedance Analyzer	X	X	X

	Trunk muscle mass(kg)	Bioelectrical Impedance Analyzer	**X**	**X**	**X**
	Appendicular lean mass(kg)	Bioelectrical Impedance Analyzer	**X**	**X**	**X**
	Body fat rate（%）	Bioelectrical Impedance Analyzer	**X**	**X**	**X**
	Fat mass (kg)	Bioelectrical Impedance Analyzer	**X**	**X**	**X**
	Trunk Fat mass (kg)	Bioelectrical Impedance Analyzer	**X**	**X**	**X**
	Visceral fat grade	Bioelectrical Impedance Analyzer	**X**	**X**	**X**
	**Comprehensive Geriatric Assessment-Questionnaire**				
	Frailty index	Questionnaire survey	**X**		**X**
	Short Form-12 Health Survey	Questionnaire survey	**X**		**X**
	Fatigue Scale-14	Questionnaire survey	**X**		**X**
	Physical Fatigue	Questionnaire survey	**X**		**X**
	Mental fatigue	Questionnaire survey	**X**		**X**
	Montreal Cognitive Assessment	Questionnaire survey	**X**		**X**
	Pittsburgh Sleep Quality Index	Questionnaire survey	**X**		**X**
	Kessler 10 scale	Questionnaire survey	**X**		**X**
**Safety**	**Tumor biomarker**				
	CEA (ng/ml)	Venous blood draw (fasted)	**X**	**X**	**X**
	AFP (ng/ml)	Venous blood draw (fasted)	**X**	**X**	**X**
	**Liver and kidney function**				
	ALT(U/L)	Venous blood draw (fasted)	**X**	**X**	**X**
	AST(U/L)	Venous blood draw (fasted)	**X**	**X**	**X**
	ALP(U/L)	Venous blood draw (fasted)	**X**	**X**	**X**
	TBA (umol/L)	Venous blood draw (fasted)	**X**	**X**	**X**
	Cys-C (mg/L)	Venous blood draw (fasted)	**X**	**X**	**X**
	UA (umol/L)	Venous blood draw (fasted)	**X**	**X**	**X**
	β2-MG (μg/L)	Venous blood draw (fasted)	**X**	**X**	**X**

	U-ALB (g/L)	Venous blood draw (fasted)	**X**	**X**	**X**
	**Routine examination of blood**				
	RBC (10^9/L)	Venous blood draw (fasted)	**X**	**X**	**X**
	WBC (10^9/L)	Venous blood draw (fasted)	**X**	**X**	**X**
	Lymphocyte absolute value (10^9/L)	Venous blood draw (fasted)	**X**	**X**	**X**
	Neutrophil absolute value (10^9/L)	Venous blood draw (fasted)	**X**	**X**	**X**
	**Adverse events**	Follow-up visit	⟶	⟶	⟶
**Dietary intake**	Habitual food and nutrient intake (g /day)	Food Frequency Questionnaire	**X**		**X**
	Accurate food and nutrient intake (g /day)	Photo-Assisted three-day 24-hour Dietary Intake Assessment	**X**		**X**

* Median DNAmAge, DNA methylation median age; HOMA-IR, Homeostasis Model Assessment of Insulin Resistance; FBG, Fasting Blood Glucose; INS, Insulin; HbA1c, Hemoglobin A1c; LDL, Low-Density Lipoprotein; TC, Total Cholesterol; TG, Triglycerides; TNFα, Tumor Necrosis Factor α; IL6, Interleukin-6; MDA, Malondialdehyde; γH2AX, γH2A Histone Family Member X; CEA, Carcinoembryonic Antigen; AFP, Alpha-Fetoprotein; ALT, Alanine Aminotransferase; AST, Aspartate Aminotransferase; ALP, Alkaline Phosphatase; TBA, Total Bile Acids; Cys-C, Cystatin C; UA, Uric Acid; β2-MG, β-2 Microglobulin; U-ALB, Urinary Albumin; RBC, Red Blood Cell Count; WBC, White Blood Cell Count.

Table S13. List of instruments and reagents.

Category	Marker	Method	Instrument/Reagent
Glucose Metabolism	Fasting Blood Glucose (FBG)	Glucose Oxidase	Fosun AU5800 Analyzer
Glucose Metabolism	Glycated Hemoglobin (HbA1c)	HPLC	Tosoh HCL-G11 HPLC Analyzer
Glucose Metabolism	Insulin	Chemiluminescent Immunoassay (CLIA)	Roche COBAS 6000 System
Lipid Profile	Total Cholesterol (TC)	Enzymatic Method	Fosun AU5800 Analyzer
Lipid Profile	LDL Cholesterol (LDL-C)	Enzymatic Method	Fosun AU5800 Analyzer
Lipid Profile	Triglycerides (TG)	Enzymatic Method	Fosun AU5800 Analyzer
Liver Function	Alanine Aminotransferase(ALT)	Enzymatic Method	Fosun AU5800 Analyzer
Liver Function	Aspartate Aminotransferase(AST)	Enzymatic Method	Fosun AU5800 Analyzer
Liver Function	Alkaline Phosphatase(ALP)	Enzymatic Method	Fosun AU5800 Analyzer
Liver Function	Albumin (Alb)	Enzymatic Method	Fosun AU5800 Analyzer
Kidney Function	Creatinine (Cr)	Enzymatic Method	Roche C701 Analyzer
Kidney Function	Blood Urea Nitrogen (BUN)	Enzymatic Method	Roche C701 Analyzer
Kidney Function	Cystatin C (Cys-C)	Enzymatic Method	Roche C701 Analyzer
Kidney Function	β2-Microglobulin (β2-MG)	Immunoassay	Siemens Equipment
Inflammatory Markers	Interleukin-6 (IL-6)	ELISA	BD Biosciences and R&D Systems
Inflammatory Markers	TNF-α	ELISA	BD Biosciences and R&D Systems
Oxidative Stress	Malondialdehyde (MDA)	TBA Method	Nanjing Jiancheng TBA Kit
Oxidative Stress	Superoxide Dismutase (SOD)	WST-1 Method	Nanjing Jiancheng WST-1 Kit
Tumor Biomarkers	Carcinoembryonic Antigen (CEA)	CLIA	Antu and Roche Immunoassay Kits
Tumor Biomarkers	Alpha-Fetoprotein (AFP)	CLIA	Antu and Roche Immunoassay Kits
Body Composition	Body Composition	Bioelectrical Impedance	Shuangjia Inbody Device
Relative Telomere Length	Relative Telomere Length	qPCR	Shanghai Yihe Biotechnology Kit

T Lymphocyte Subsets	T Lymphocyte Subsets	Flow Cytometry	Bio-Rad ZE5 Flow Cytometer
Additional Markers	Phosphorylated Histone H2AX (p-H2AX)	ELISA	R&D Systems DuoSet ELISA Kit
Additional Markers	IGF-I	ELISA	R&D Systems DuoSet ELISA Kit

Venous Blood Draw (Fasted)

1. Telomere Length (T/S ratio): Relative telomere length was determined via quantitative PCR (qPCR), comparing telomeric DNA amplification to a reference gene. Kits from Shanghai Yihe Biotechnology were used for the assay.
2. DNA Methylation Clock: This study employed whole-genome bisulfite sequencing (WGBS) to analyze DNA methylation levels in blood samples. Following DNA extraction from clotted blood, libraries were constructed using the ZYMO EZ DNA Methylation-Gold kit, followed by quality assessment and high-throughput sequencing. Data processing utilized SOAPnuke and bismark software to filter low-quality reads, align the clean reads to the reference genome, extract methylation information, and generate methylation coverage maps for further analysis.
3. Glycemic Metabolic Profile: Fasting blood glucose (FBG) was measured using the glucose oxidase method, which quantifies glucose based on its enzymatic conversion to gluconic acid and hydrogen peroxide, with subsequent colorimetric detection. Analyses were performed using the Fosun AU5800 automated biochemical analyzer. Glycated hemoglobin (HbA1c) was determined by high-performance liquid chromatography (HPLC) with the Tosoh HCL-G11 HPLC analyzer. Insulin levels were measured by chemiluminescent immunoassay (CLIA) on the Roche COBAS 6000 system, where the intensity of emitted light is proportional to insulin concentration.
4. Lipid Profile: Total cholesterol (TC), low-density lipoprotein cholesterol (LDL-C), and triglycerides (TG) were assessed via enzymatic methods, with enzymatic reactions producing colorimetric products proportional to lipid concentrations. These were analyzed using the Fosun AU5800 automated biochemical analyzer.
5. T Lymphocyte Subsets: T lymphocyte subsets (CD3, CD4, CD8,) were assessed by flow cytometry using a Bio-Rad ZE5 flow cytometer and BD Pharmingen antibodies, with fluorescence-labeled antibodies targeting specific cell surface markers.
6. Inflammatory Markers: Inflammatory Markers, including IL-6, TNF-α were measured using a QAH-CUST chip based on the Quantibody® Custom Array protocol from RayBiotech.
7. Oxidative Stress Markers: Malondialdehyde (MDA), a marker of lipid peroxidation, was determined using the thiobarbituric acid (TBA) method.

Superoxide dismutase (SOD) activity was measured via the WST-1 method, while glutathione peroxidase (GSH-Px) was assayed by colorimetric methods. All oxidative stress markers were analyzed with kits from Nanjing Jiancheng Bioengineering Institute.

8. Phosphorylated histone H_2AX (p-H_2AX) were measured via ELISA using R&D Systems DuoSet ELISA kits.
9. Liver and Kidney Function: Liver enzymes, including alanine aminotransferase (ALT), aspartate aminotransferase (AST), alkaline phosphatase (ALP), albumin (Alb), and total bile acids (TBA), were measured by enzymatic assays. Gamma-glutamyl transferase (GGT) was assessed using the GPNA substrate method, with Fosun AU5800 analyzers and Fosun diagnostic kits. Renal function markers such as creatinine (Cr), blood urea nitrogen (BUN), cystatin C (Cys-C), and β2-microglobulin (β2-MG) were quantified using enzymatic and immunoassay methods with Roche C701 and Siemens equipment.
10. Tumor Biomarkers: Carcinoembryonic antigen (CEA) and alpha-fetoprotein (AFP) were measured via CLIA, where emitted light is proportional to marker concentrations. Assays were conducted using Antu and Roche immunoassay kits.

Questionnaire-Based Assessments

1. Frailty Index: The Frailty Index was assessed through a self-reported questionnaire, comprising 28 baseline health variables. These variables included chronic conditions, functional impairments, symptoms, and physical measurements (e.g., body mass index). Each variable was coded as either 0 (no deficit) or 1 (deficit present), and the final frailty score was calculated as the mean of all variables, with a score ranging from 0 to 1. Higher scores indicate greater frailty. This method has been validated in multiple epidemiological studies.
2. Short Form-12 Health Survey (SF-12): The SF-12 is a standardized self-reported questionnaire designed to assess health-related quality of life, encompassing physical and mental health domains. It consists of 12 items, with scores ranging from 0 to 100. Higher scores reflect better perceived health and quality of life. The SF-12 is widely used in large-scale health studies due to its brevity and reliability for quickly assessing overall health status.3
3. Montreal Cognitive Assessment (MoCA): The MoCA is a screening tool for mild cognitive impairment, assessing multiple cognitive domains including attention, memory, language, visuospatial abilities, and executive function. It consists of 30 items with a total score of 30 points, where scores below 26 indicate potential cognitive impairment. MoCA is highly sensitive to early cognitive decline and has been widely validated.
4. Pittsburgh Sleep Quality Index (PSQI): The PSQI assesses sleep quality over the past month, consisting of 19 items across 7 domains, such as sleep latency, duration, efficiency, and daytime dysfunction. The total score ranges from 0 to 21, with higher scores indicating poorer sleep quality. A score above 5 suggests significant sleep disturbances. PSQI is widely used in both sleep research and clinical assessments.

5. Fatigue Scale-14: This scale evaluates both physical and mental fatigue through 14 items. Participants rate their fatigue levels, with a total score ranging from 0 to 56. Higher scores indicate greater fatigue severity. The Fatigue Scale-14 has been widely used in chronic disease populations, with established validity and reliability.
6. Kessler Psychological Distress Scale (K10): The K10 assesses psychological distress experienced over the past four weeks. It consists of 10 items, with a scoring range of 10 to 50. Higher scores indicate higher levels of psychological distress. The K10 is primarily used for screening anxiety and depression in both clinical and epidemiological settings, offering a concise yet effective tool for mental health assessment.
7. Dietary Intake Assessment: Dietary intake was assessed using a combination of the 24-hour dietary record (24HR) method and the Food Frequency Questionnaire (FFQ). The 24HR method utilized a photo-assisted approach [1], where participants were asked to take photos of their three meals over three consecutive days. The dietary intake was later calculated uniformly by investigators. In addition, the FFQ questionnaire was used to assess habitual dietary intake, particularly focusing on nucleotide-rich foods and seasonings. The FFQ captured habitual dietary intake, particularly focusing on nucleotide-rich foods and seasonings. This combined approach provided a detailed assessment of both short-term and long-term dietary patterns, enhancing the accuracy and comprehensiveness of the dietary data.

References

[1] Fan R, Chen Q, Song L, et al. The Validity and Feasibility of Utilizing the Photo-Assisted Dietary Intake Assessment among College Students and Elderly Individuals in China [J]. Nutrients, 2024, 16(2): 211.

Performance-Based Tests

1. Grip Strength: Handgrip strength is measured using a dynamometer to assess muscle strength. The dominant hand is used for the test, and the participant performs two trials, with the highest value (kg) recorded as the final result.
2. Gait Speed (6 meters): The time taken for the participant to walk 6 meters at a normal pace is measured. Marked distances on the ground are used to calculate gait speed (m/s), reflecting functional mobility.
3. Timed Up and Go (TUG): The participant stands up from a chair, walks 3 meters, turns around, and sits back down. The total time (seconds) is recorded, providing an assessment of the integrated ability to stand, walk, and turn.
4. Tinetti Score: This assessment includes two parts: balance and gait. It evaluates the participant’s performance in both areas, with scores assigned based on

their ability. The total score is 28 points, with higher scores indicating better balance and gait function.

5. Short Physical Performance Battery (SPPB): This battery includes three components: balance tests, gait speed (4 meters), and sit-to-stand tests. The balance tests assess the ability to maintain different standing positions, the gait test measures time to walk 4 meters, and the sit-to-stand test evaluates the time to complete five repeated stands. The total score is 12 points, used to assess lower extremity function.

Anthropometric Measurements

Anthropometric measurements were taken using a flexible tape measure to accurately assess body circumferences. Standardized protocols were followed to measure specific circumferences, including waist, hip, neck, mid-arm, and lower leg circumferences, ensuring consistency and precision in the assessment.

Non-invasive Instrumental Measurements

Body composition: it was analyzed using bioelectrical impedance analysis (BIA), a validated method for estimating the distribution of fat mass and lean mass in the human body. BIA was employed to measure whole-body fat mass, lean mass, body fat percentage, trunk fat, and muscle mass, along with other key body compartments. Measurements were conducted following established guidelines to ensure consistency and reliability in body composition evaluation.
